# Ideological Cues, Partisanship, and Prejudice Against LGBTQ Judges

**DOI:** 10.1093/poq/nfaf064

**Published:** 2026-03-03

**Authors:** Andrew R Stone, Tony Zirui Yang

**Affiliations:** Assistant Professor, Department of Political Science, University of Mississippi, University, MS, US; Assistant Professor, Department of Political Science, Emory University, Atlanta, GA, US; Postdoctoral Prize Research Fellow in Politics, Nuffield College, University of Oxford, Oxford, UK

## Abstract

How does the gender and sexual identity of a prospective judge shape public support for their nomination? We build upon recent scholarship on instrumental inclusivity and argue that, after accounting for nominee ideology, Americans of all partisan stripes will penalize LGBTQ nominees. Using a conjoint experiment, we randomly vary a prospective Biden US Supreme Court nominee’s gender and sexual identity. Crucially, we also randomize the nominee’s ideology, enabling us to disentangle LGBTQ identity from the ideological signal it sends and differentiate between genuine and instrumental support for LGBTQ nominees. Contrary to recent findings suggesting that Democrats reward minority judges, we find that respondents from both parties penalize LGBTQ nominees. The magnitude of these effects—roughly 14 percentage points for transgender nominees and 8 percentage points for gay or lesbian nominees—is considerable and second only to shared partisanship. Our study underscores that ideological alignment does not necessarily foster genuine inclusivity for LGBTQ individuals and highlights the persistent challenges of representation for marginalized groups in an era of polarized judicial nominations.

The American judiciary has played an active and critical role in one of the most contentious areas of policymaking in contemporary American politics: the promotion and restriction of LGBTQ rights (Bailey et al. 2025). At the same time, the demographic characteristics of judicial nominees, including gender and sexual identity, have gained greater political attention.^1^ These traits allow politicians to appeal to core political constituencies and mobilize support for nominees (Kaslovsky, Rogowski, and Stone 2021), shape confirmation hearings (Boyd, Collins, and Ringhand 2025), and influence the substantive decisions courts make on issues important to underrepresented groups (Boyd, Epstein, and Martin 2010; Kastellec 2013).

A key player in the nomination and confirmation process is the American public. Public attitudes toward nominees shape how politicians behave on judicial nominations (Kastellec, Lax, and Phillips 2010) and impact how Americans evaluate politicians (Bass, Cameron, and Kastellec 2022) and the judiciary as an institution (Glick 2023). In this way, public attitudes toward nominees shape the choices of presidents and senators and thus determine the descriptive traits of judges who end up on the bench.

In this study, we focus on how the gender and sexual identity of prospective judicial nominees impacts their public support, an area largely overlooked in previous research. A pioneering study by Bracic et al. (2023) finds that Democrats are more trusting of gay than straight judges, while Republicans exhibit the opposite pattern. Consistent with research on LGBTQ candidates (Jones and Brewer 2019; Magni and Reynolds 2021), Bracic et al. (2023) also find that both Republicans and Democrats view gay judges as more liberal than their straight counterparts. This study does not, however, disentangle perceptions of ideology from the sexuality of the judge and thus cannot speak to an important theoretical discussion in the study of attitudes toward LGBTQ politics: whether individuals genuinely support LGBTQ politicians or whether their identity provides an instrumental reason to do so (Kwon, Scarborough, and Taylor 2023; Turnbull-Dugarte and López Ortega 2024).

We build upon scholarship of instrumental inclusivity (Turnbull-Dugarte and López Ortega 2024) to theorize that Democrats’ support for LGBTQ judges is driven by ideological motivations (Sen 2017), revealing prejudice when ideological and partisan considerations are accounted for. To test this, we conduct a conjoint experiment to assess Americans’ support for prospective Biden Supreme Court nominees who are lesbian, gay, or transgender. We randomize nominee ideology to isolate the independent effects of these traits.

Our results reveal a substantial penalty for LGBTQ nominees among Democrats and Republicans alike, with only shared partisanship having a larger effect. Across all respondents, the penalty for transgender nominees (about 13.8 percentage points) is larger than for gay and lesbian nominees (8.1 p.p.); these penalties benchmark closely to the context of the US House (Magni and Reynolds 2021). We thus find limited evidence of genuine support for LGBTQ judges after disentangling perceptions of ideology from the traits themselves. We also find a “double penalty” for gay and transgender nominees and that respondents penalize women LGBTQ nominees more than men, in line with scholarship on public reactions to politicians with intersectional minority identities (Crenshaw 1991; Hancock 2007; López Ortega and Radojevic 2025).

Our study makes a significant contribution to understanding prejudice against LGBTQ politicians, judges, and individuals. We extend the theoretical framework of instrumental support for marginalized groups beyond ethnicity and nationalism to partisanship and ideology (Sen 2017; Kwon, Scarborough, and Taylor 2023; Turnbull-Dugarte and López Ortega 2024), revealing a sizable and widespread bias against LGBTQ judges. Although we focus on attitudes toward judges, in the conclusion we consider how our ideological instrumentality argument may apply to perceptions of other political figures, such as legislators and executives (Magni and Reynolds 2021; Thompson 2023), or policy areas such as adoption or education (Turnbull-Dugarte and López Ortega 2024; López Ortega and Blanco Forthcoming).

## Instrumental Support for LGBTQ Judges

Despite decades of progress that has enhanced social acceptance of LGBTQ individuals and politicians (Ayoub 2015; Ayoub and Garretson 2017; Magni and Reynolds 2018; Abou-Chadi and Finnigan 2019), prejudice remains prevalent (Magni and Reynolds 2021; Thompson 2023). A growing body of research attributes this persistent bias to political ideology and partisanship. In the United States, scholars find that Democrats and liberals tend to be more supportive of LGBTQ individuals, politicians, and judges, whereas Republicans and conservatives are more likely to express opposition (Jones and Brewer 2019; Bracic et al. 2023). Further, scholarship shows that LGBTQ identity signals a politician’s liberal ideology (Jones and Brewer 2019; Bracic et al. 2023), much like other characteristics such as race (Sen 2017; Harrison, Michelson, and Perry 2024). These dynamics raise an important theoretical question: To what extent are liberals *genuinely* supportive of LGBTQ judges versus viewing their identity as an *instrumental* factor to secure policy representation (Turnbull-Dugarte and López Ortega 2024)?

Identifying whether support for LGBTQ individuals is instrumental or genuine is crucial, as instrumental support is opportunistic and conditional, offering little in terms of reducing the persistence of LGBTQ prejudice. In their groundbreaking study, Turnbull-Dugarte and López Ortega (2024) demonstrate that conservative and nationalistic individuals opposed to immigration strategically adopt pro-LGBTQ attitudes to align with their co-ethnic in-group. We argue that this instrumental inclusivity theory extends beyond right-wing homonationalism to left-wing support for LGBTQ judges. In short, what may appear as growing social acceptance of LGBTQ issues may be driven by instrumental liberalism.

Citizens have policy preferences and should be willing to align themselves with ideological in-groups. Left-leaning individuals have a strong incentive to strategically support LGBTQ judges, as doing so can advance liberal policy outcomes. Consequently, when ideological instrumental incentives are accounted for, liberals may not be as supportive of LGBTQ politicians and judges as existing studies suggest. Teasing out ideological cues from LGBTQ identity is thus important for assessing the independent effects of gender and sexual identity.

Scholarship on the courts provides support for our argument. The public values judges who share their political affiliations (Bartels and Johnston 2012) and evaluates the courts through the lens of identity, such as judge race and gender (Sen 2017; Zilis 2021; Ono and Zilis 2022). These patterns parallel the finding of Bracic et al. (2023) for LGBTQ judges. When ideological cues are not isolated from gender and sexual identity, LGBTQ individuals are more likely to be considered liberal and thus in-group by left-leaning citizens and out-group by those on the right. This should bolster support for LGBTQ judges among liberals and decrease support among conservatives.

After accounting for ideology and thus disentangling the effect of LGBTQ identity from its ideological signal, what should remain is the individual’s attitudes toward LGBTQ traits themselves. Scholarship illustrates how racial prejudice exists and shapes the political behavior of individuals across the political spectrum (e.g., Krupnikov and Piston 2015; Hooghe and Dassonneville 2018).^2^ Given extant prejudices that persist across partisan groups against LGBTQ individuals, we expect a similar penalty for judges with LGBTQ traits (Jones et al. 2018; Magni and Reynolds 2021; Thompson 2023). These negative effects should appear stronger for transgender judges, when compared to gay judges, given the higher level of prejudice against these individuals (Lewis et al. 2017; Magni and Reynolds 2021). Moreover, individuals with multiple intersecting minority traits (Hancock 2007) can receive additional backlash (López Ortega and Radojevic 2025). We expect that stronger effects may emerge for LGBTQ judges with other minority traits.

Support for LGBTQ judges provides an ideal test case for the theory of instrumental ideological inclusivity, as the federal judicial nomination and confirmation process has become increasingly polarized along political lines. Developments such as the removal of the filibuster and President Obama’s failed nomination of Merrick Garland exemplify this trend (Boyd, Lynch, and Madonna 2015; Cameron and Kastellec 2023). Public attitudes toward the judiciary have also grown more polarized (Levendusky et al. 2024), with elite cues playing a significant role in shaping these evaluations (Rogowski and Stone 2021). As with other institutions, Americans demand both policy and descriptive representation on the bench (Scherer and Curry 2010; Bartels and Johnston 2012; Kaslovsky, Rogowski, and Stone 2021). Given the parallels between judicial and electoral politics, our argument likely extends beyond judges to LGBTQ candidates more broadly.^3^

## Experimental Design

To estimate the effect of a Supreme Court nominee’s gender and sexual identity on public evaluations, we employ a preregistered conjoint experiment.^4^ The design presents respondents with a hypothetical profile of a Supreme Court nominee that randomly varies several nominee attributes, including LGBTQ identity and ideology. This design has three important methodological benefits. First, we control for a nominee’s ideology, allowing us to isolate the independent effect of LGBTQ identity and test the extent to which Americans exhibit genuine support for these nominees. Second, it minimizes concerns about social desirability bias regarding attitudes toward LGBTQ individuals due to the presence of other traits apart from gender and sexual identity. Finally, this setup realistically reflects the information available to Americans when evaluating judicial nominees and has been used to study attitudes toward prospective nominees (Sen 2017; Rogowski and Stone 2021).

We draw upon a sample of 1,249 American adult respondents on the CloudResearch Connect online platform from December 22, 2023, to January 4, 2024. To provide an accurate picture of the preferences of the American population, we employed a quota to match US Census demographics on race, ethnicity, gender, and age. We also quota-sampled on partisanship to recruit a sufficient number of Republicans and Democrats, with leaners coded as partisans in main analyses. We do not employ weighting in any analyses. Over 95 percent of respondents passed our attention check (see Appendix A for details).

Our design was straightforward. Respondents were presented with a hypothetical profile of a Biden Supreme Court nominee and then evaluated their support for the nominee. Respondents completed two nominee evaluations, one at a time; our analyses cluster standard errors at the respondent level. Using Biden as the nominating president allows us to hold constant respondent views of the nominating president’s ideology. The profiles contained several randomized demographic characteristics and personal traits that are common points of discussion during nominations, including age, race/ethnicity, current job, and law school background. Half of respondents also received one of four statements endorsing the nominee from Biden. We present these varying traits in Appendix table A1.

Our main treatment variables are the nominee’s gender identity (man, woman, transgender man, or transgender woman) and sexual orientation (straight or gay/lesbian). Because the nominee’s gender and sexual identities may cue their political ideology, we also randomized the nominee’s ideology (very liberal, liberal, somewhat liberal, or moderate); such a range reflects the plausible spectrum of Democratic Supreme Court nominees. This allows us to estimate the impact of sexual and gender identity on respondent evaluations while holding ideology constant.

After reviewing a nominee profile, respondents rated their nominee support on a five-point scale, which we recode into a binary variable, with “strongly support” and “somewhat support” coded as 1 and other options as 0.^5^ For additional survey information, see Appendix A.

## Results

**Figure 1.**
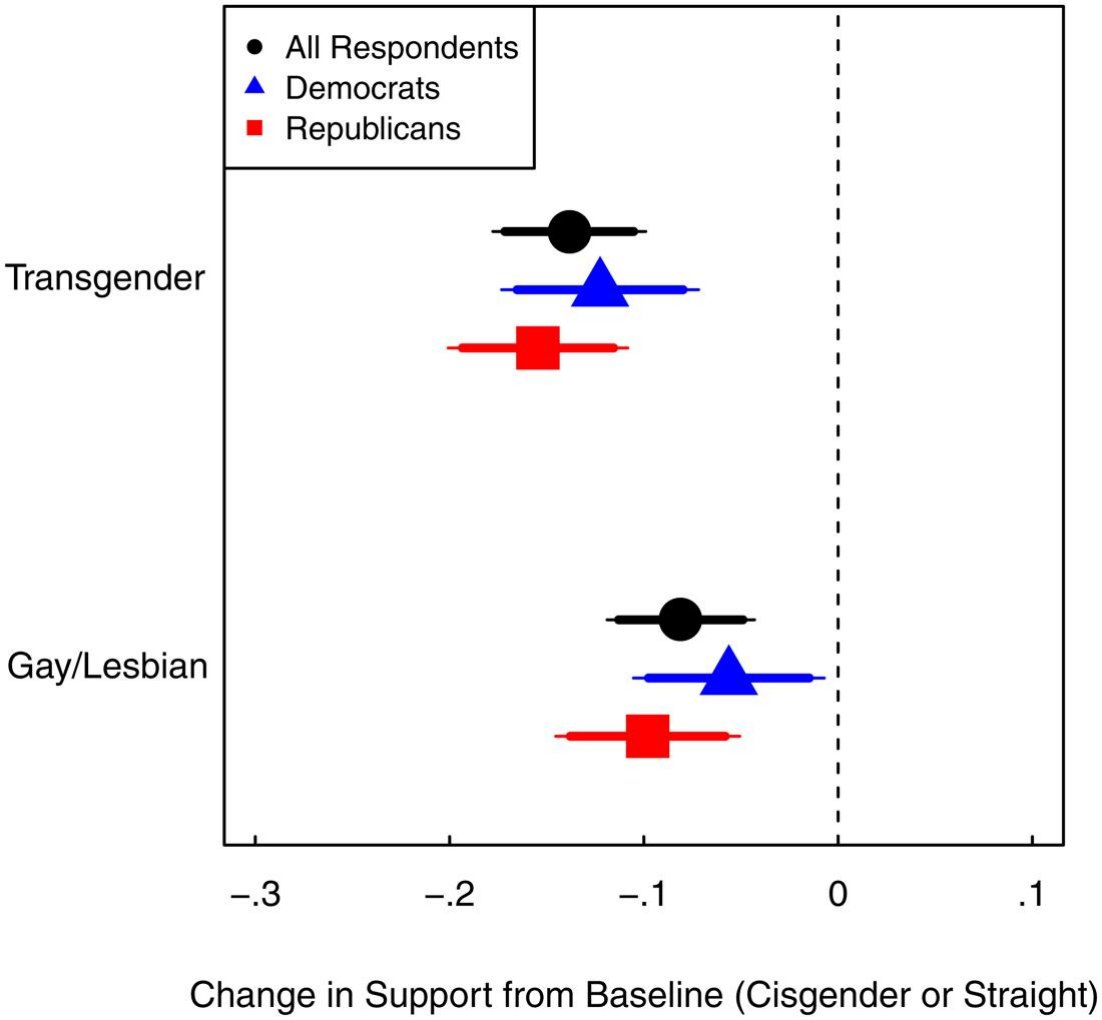
All partisans penalize LGBTQ Supreme Court nominees. Estimated treatment effects (AMCE) of a nominee’s transgender or gay/lesbian identity (as compared to the baseline of cisgender or straight) on binary support for the nominee for all respondents, Democrats, and Republicans. There are 90 and 95 percent confidence intervals plotted for each estimate; standard errors are clustered by respondent. Leaners are coded as partisans. Full results are available in Supplementary Material figures D.1 and D.2.

Figure 1 presents the average difference in binary support for nominees who are transgender or gay compared to their cisgender or straight counterparts, averaged across all other nominee characteristics (the Average Marginal Component Effect). We present separate effects for all respondents, Democrats, and Republicans. When averaging across all respondents, prospective nominees who identify as gay or lesbian experience an 8.1 percentage point (*p *< 0.001) decrease in support compared to straight nominees, and transgender nominees face a 13.8 percentage point (*p *< 0.001) decrease in comparison to cisgender nominees. Similar to previous studies, the negative effect observed for transgender individuals is significantly larger than that of gays and lesbians (*p *=* *0.037). These findings are robust across various alternative analyses (see Supplementary Material section E for a full discussion). Our findings provide clear evidence that LGBTQ nominees receive less public support than their straight and cisgender counterparts, with transgender individuals facing the greatest backlash.

Crucially, we find consistently lower support for LGBTQ nominees across partisan groups. Although baseline support for Biden’s prospective nominee is considerably higher among Democrats than Republicans (by 53.5 p.p.), the penalty for LGBTQ nominees is evident across all partisan groups. For instance, the decrease in support for transgender nominees is 15.4 percentage points (*p *<* *0.001) among Republicans and 12.3 percentage points (*p *<* *0.001) among Democrats. These treatment effects are not distinguishable from one another (*p *=* *0.361). Once we isolate ideological cues from gender and sexual identity, we uncover a consistent penalty for LGBTQ individuals across partisan lines (c.f. Bracic et al. 2023).

The magnitude of the negative treatment effects of gender and sexual identity is particularly noteworthy when juxtaposed with the results associated with other attributes (Supplementary Material figure D.1). The only other attribute yielding statistically and substantively significant results comparable to gender and sexual identity is the nominee’s law school. Judges who graduated from non–top 100 schools received 12.3 percentage points (*p *<* *0.001) lower support in comparison to elite Ivy graduates. Strikingly, the effects of gender and sexual identity overshadow the effect of ideology. When disaggregating by partisanship (Supplementary Material figure D.2), Republican respondents favor moderate nominees over very liberal nominees by 14.0 percentage points (*p *<* *0.001). In contrast, the effects of ideology for Democrats are substantively small and statistically insignificant, with, for example, a 3.2 percentage point (*p *=* *0.395) preference for very liberal over moderate nominees.

**Figure 2.**
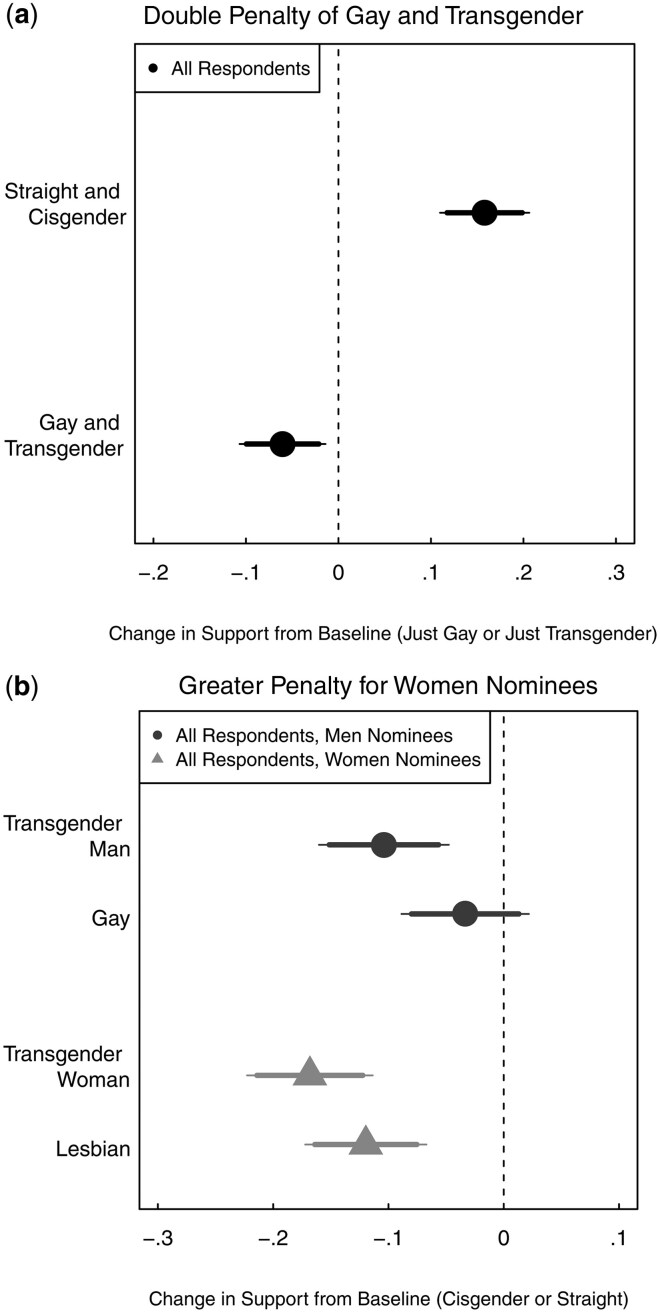
Intersectional traits and support for LGBTQ nominees. Panel (a) shows estimated treatment effects (AMCE) on binary support of judges who are transgender and gay/lesbian (bottom point) or judges who are cisgender and straight (top point) as compared to judges who are transgender or gay/lesbian (baseline). Panel (b) shows estimated treatment effects of men (top points) and women (bottom points) nominees who are gay and transgender. There are 90 and 95 percent confidence intervals plotted for each estimate; standard errors are clustered by respondent. Estimates pool all respondents. Full results are available in Supplementary Material figures D.3, D.4, and D.5.

We also assess, first, whether candidates who are both gay and transgender face an additional penalty, and second, whether our findings differ for women and men nominees. Both analyses speak to how the public reacts to the intersection of multiple minority traits (Crenshaw 1991; Hancock 2007; Cassese 2019). To conduct the first analysis, we set “just gay or just transgender” as the baseline category and assess how respondents differentially evaluate nominees with both (or neither) of these traits. For the second, we divide the sample based on nominee gender and conduct separate analyses for men and women nominees.

In figure 2a, we illustrate that judges who are both transgender and gay pay a greater penalty (6.0 p.p. lower support, *p *=* *0.011) than judges with just one of those traits. In figure 2b, we illustrate that the magnitude of the negative effect for both transgender and gay/lesbian status is larger for women (16.8 and 12.0 p.p., respectively) than men (10.4 and 3.3, respectively); this difference is statistically significant for gay/lesbian (*p *=* *0.027) but not transgender nominees (*p *=* *0.108). These findings highlight that, as in other political contexts, LGBTQ judicial nominees with intersectional identities face additional challenges in garnering public support.^6^

## Discussion and Conclusion

By extending recent scholarship on instrumental inclusivity beyond right-wing homonationalism to left-wing support for LGBTQ judges (Turnbull-Dugarte and López Ortega 2024), we demonstrate that, after accounting for the ideology of judges, both Democrats and Republicans discriminate against LGBTQ judicial nominees. This challenges the common assumption that left-leaning individuals and parties are inherently more tolerant toward gender and sexual minorities and underscores the need to distinguish between strategic and genuine support for marginalized groups.

We conclude with a consideration of three important implications of our study. First, we consider the bipartisan nature of our findings. Does this suggest that Democrats and liberals falsify a true dislike of LGBTQ individuals when publicly advocating for LGBTQ rights and inclusion? We are hesitant to draw this conclusion. We see an important theoretical distinction between concealing one’s true preferences due to fear of public sanction and instrumental support. Democrats and liberals may truthfully support LGBTQ rights and representation, but their support could be driven more by self-interest, such as advancing liberal policy outcomes, than by genuine altruistic acceptance. Nevertheless, future studies should assess these dynamics, especially as our conjoint design minimizes the potential for social desirability bias and thus our ability to study conditions under which preference falsification may emerge.

Second, we consider the generalizability of our findings. We focus on LGBTQ judges, given the active role of the judiciary in shaping LGBTQ rights (Bailey et al. 2025), the role descriptive traits play in shaping judge behavior (Boyd, Epstein, and Martin 2010; Kastellec 2013), and the political importance to the public of contemporary judicial nominations (Bartels and Johnston 2012; Bass, Cameron, and Kastellec 2022). We expect that our theoretical framework of instrumental ideological inclusivity should largely apply to other politicians who require direct public approval, such as members of Congress and executives.^7^ However, extending our theory to public support for specific policies—such as adoption, surrogacy, immigration, or education—may be more complex. In these contexts, the instrumental ideological incentives to elect a representative or confirm a judge to secure desired ideological policy are not present. More research is needed to disentangle the specific calculations at play in these contexts.

Finally, we highlight the challenges marginalized groups face in achieving representation within the judiciary and American political institutions, especially for nominees with intersecting minority traits. As most Americans are cisgender and straight, our results are in line with scholarship illustrating that Americans of all partisan stripes desire judges who share their descriptive traits (Scherer and Curry 2010; Kaslovsky, Rogowski, and Stone 2021). Our findings help explain the underrepresentation of LGBTQ individuals on the bench given the public opinion pressures politicians face on judicial nominations (Bass, Cameron, and Kastellec 2022). Like Bracic et al. (2023), we see our findings as illustrative that LGBTQ judges may seek to avoid public discussion of their identity to avoid potential backlash; in contrast, our theory and empirical approach highlights that this disincentive likely exists for potential nominees across the political spectrum. Nevertheless, Presidents Obama, Trump, and Biden have all nominated openly gay judges. This illustrates that LGBTQ jurists still have a viable pathway to the federal bench, though none of these were Supreme Court nominees—who command considerably greater scrutiny than lower court nominees—and presidents have entirely avoided nominating openly transgender judges. While diversifying the judiciary would likely enhance LGBTQ substantive representation, public resistance creates a challenging path for prospective LGBTQ judges.

## Supplementary Material

nfaf064_Supplementary_Data

## Data Availability

Replication data and documentation are available at https://doi.org/10.7910/DVN/CDLVDH.

## Footnotes

1 For instance, 5.1 percent (12 total, a record) of President Biden’s nominees were LGBTQ; the American LGBTQ population is approximately 7.6 percent (Chavez and Choi 2024; Raymond 2024).

2 See Supplementary Material section F.1 for more discussion.

3 However, the public also expects judges to prioritize legalistic principles (Rivero and Stone 2025), distinguishing the judiciary from other institutions.

4 See Supplementary Material section C for pre-analysis plan discussion.

5 We asked, “On a scale from strongly oppose to strongly support, where would you place your level of support for this nominee?” Response options were strongly oppose/somewhat oppose/neither oppose nor support/somewhat support/strongly support. Two respondents who did not answer this question are dropped from analyses (see Appendix A).

6 When subsetting to cisgender, straight nominees (Supplementary Material figure E.10), women are viewed 6.9 p.p. more favorably than men (though we cautiously interpret this result as *p* = 0.084). This aligns with findings on favorability toward women in conjoints (Schwarz and Coppock 2022) and suggests that multiple minority traits intersect to penalize politicians in non-additive ways (Hancock 2007).

7 See Supplementary Material section F.2 for more discussion.

## Appendix A. AAPOR-Required Disclosure Elements

**Data Source and Data Collection Strategy:** We employ survey data collected via a survey we administered on the CloudResearch Connect online platform.

**Research Sponsor and Conductor:** The research was sponsored by resources provided to Tony Zirui Yang from Washington University in St. Louis. The research was conducted by the authors.

**Measurement Tools/Instruments:** The survey began with an informed consent screen. Then, respondents were asked four factual Supreme Court knowledge questions:

- Do you know if the U.S. Supreme Court can declare an act of Congress unconstitutional, or does it not have this power?
  - Can declare an act unconstitutional/Cannot declare an act unconstitutional/Don’t know
- Some judges in the U.S. are elected; others are appointed. Do you happen to know if the Justices of the U.S. Supreme Court are elected or appointed?
  - Elected/Appointed/Some are elected and some are appointed/Don’t know
- Some judges in the U.S. serve for a set number of years; others serve a life term. Do you happen to know whether the Justices of the U.S. Supreme Court serve for a set number of years or whether they serve a life term?
  - Set number of years/Life term/Some serve a set number of years and some serve a life term/Don’t know
- Which of the following people does not currently serve on the U.S. Supreme Court?
  - Clarence Thomas/Samuel Alito/Anthony Kennedy/Elena Kagan/Sonia Sotomayor/John Roberts

Then, respondents were presented with an attention check that read as follows:

- What news people watch affects their judgment on many issues. However, in this question, we only want to test whether you pay attention to the questions. Hence, regardless of what you are interested in, please choose Economic News and Sports News.
  - Political News/Local News/International News/Economic News/News Interviews/Investigative Journalism/Entertainment News/Technology News/Stock Market News/Sports News/All of the above/None of the above

Then, respondents were presented with the first hypothetical nominee profile. They received the following introductory prompt and the profile, where each nominee had one of the randomly assigned characteristics displayed in Table A1 apart from the statement from Biden, which was randomly assigned to be shown to only half of the respondents:

**Table A1.** Characteristics of hypothetical Supreme Court judges.

Age	(a) 45; (b) 55; (c) 65
Race/ethnicity	(a) Black; (b) Asian; (c) Hispanic
Law school	(a) Elite law school at an Ivy League university; (b) Well-regarded law school at a large public university; (c) Law school not ranked in the top 100 law schools
Current job	(a) US district court judge; (b) Public defender; (c) Law professor at a top law school; (d) Corporate defense attorney
Political views	(a) Very liberal; (b) Liberal; (c) Somewhat liberal; (d) Moderate
Gender	(a) Man; (b) Transgender man; (c) Woman; (d) Transgender woman
Sexual orientation	(a) Straight; (b) Gay/lesbian
Statement from Biden (half of respondents)	(a) “This nominee has an outstanding legal record and is well-qualified to serve on the federal judiciary.”; (b) “This nominee will be a principled progressive voice on the federal judiciary.”; (c) “This nominee will contribute to a diverse judiciary and make the federal judiciary look more like America.”; (d) “This nominee will provide much-needed representation in the federal judiciary to a community historically underrepresented on the bench.”

*Note*: One value from each attribute was randomly assigned to respondents for each hypothetical judge, apart from the Biden statement, which was randomly assigned to half of the respondents. If respondents were randomized into receiving a judge with gay or lesbian sexual orientation, the word displayed matched the judge’s gender identity.

- Suppose that next month, a vacancy arises on the U.S. Supreme Court and President Biden nominates the following individual to the Court:

Then, respondents were asked to evaluate their support for the nominee:

- On a scale from strongly oppose to strongly support, where would you place your level of support for this nominee?
  - Strongly oppose/somewhat oppose/neither oppose nor support/somewhat support/strongly support

Then, respondents were asked to evaluate the legitimacy of the judiciary if the nominee were to be on the Court. They were first presented with the following prompt:

- Suppose this nominee was confirmed and began serving as a judge on the U.S. Supreme Court. Please indicate whether you would agree or disagree with the following, keeping in mind your evaluation of the nominee:

Then, respondents answered five legitimacy questions. All response options were a five-point measure of agreement (Strongly agree/somewhat agree/neither agree nor disagree/somewhat disagree/strongly disagree) The wording of the Supreme Court legitimacy questions are as follows:

- If the U.S. Supreme Court started making a lot of decisions that most people disagree with, it might be better to do away with the Supreme Court altogether.
- I would trust the U.S. Supreme Court to make decisions that are right for the country as a whole.
- I would support removing judges from their position on the U.S. Supreme Court if they consistently made decisions at odds with what a majority of the people want.
- The U.S. Supreme Court will have become too mixed up in politics.
- The U.S. Supreme Court will have become too independent and should be seriously reined in.

Then, respondents received a second nominee profile. They received the following prompt:

- Now, suppose that next month, a vacancy arises on the U.S. Supreme Court and President Biden nominates the following individual to the Court:

The survey then proceeded in the same manner as described above.

Finally, respondents were asked to answer questions measuring their traits and demographic information:

- How much school or college have you completed?
  - Some high school, or less/High school graduate or GED/Some college, no 4-year degree/College graduate/Post-graduate degree
- Generally speaking, do you think of yourself as a Republican, Democrat, Independent, or something else?
  - Republican/Democrat/Independent/Other
    - If Republican: Would you call yourself a strong Republican, or not a very strong Republican?
      - Strong Republican/Not very strong Republican
    - If Democrat: Would you call yourself a strong Democrat, or not a very strong Democrat?
      - Strong Democrat/Not very strong Democrat
    - If Independent or Other: Do you think of yourself as closer to the Republican or Democratic Party?
      - Republican/Democratic/Neither
- How would you describe your political views?
  - Very liberal/Somewhat liberal/Moderate/Somewhat conservative/Very conservative
- Which best describes your total annual household income? If you’re not sure, give an estimate.
  - Less than $25,000/$25,000 to $50,000/$50,000 to $75,000/$75,000 to $100,000/$100,000 to $200,000/$200,000 or more
- Which best describes your race? (Select all that apply)
  - White/Black or African American/Hispanic or Latino/Asian/American Indian or Alaska Native/Native Hawaiian or Pacific Islander/Other
- What sex were you assigned at birth, on your original birth certificate?
  - Male/Female
- What is your current gender identity?
  - Man/Woman/Transgender man/Transgender woman/Do not identify as man, woman, or transgender
- Do you think of yourself as:
  - Straight/Gay or lesbian/Bisexual/Do not identify as straight, gay or lesbian, or bisexual

**Population Under Study:** Our study population is American adults; we drew our study’s participants from the pool of American adults active on the CloudResearch Connect platform at the time of our survey.

**Methods Used to Generate and Recruit the Sample:** The sample of 1,250 respondents comes from the pool of American adults active on the CloudResearch Connect platform at the time of our survey. This is a non-probability sample. The platform is an opt-in platform. We employed the CloudResearch built-in census match template to quota-target respondents to mirror the makeup of the US population on age, race, ethnicity, and gender. These led us to quota-target 625 men and 625 women; 275 people aged 18–29, 325 aged 30–44, 325 aged 45–59, and 325 aged 60–99; 200 individuals of Hispanic origin and 1,050 individuals of non-Hispanic origin; and 975 White respondents, 175 Black respondents, and 100 respondents of other races. We also quota-sampled based upon respondent political party to secure 500 Democrats, 500 Republicans, and 250 other respondents. Respondents were paid $1.20 for completing the survey. Payment is provided through the CloudResearch platform; respondents can withdraw their earnings via PayPal, bank transfer, or Amazon gift cards.

**Method(s) and Mode(s) of Data Collection:** All survey responses were gathered on the web using Qualtrics. All surveys were conducted in English. The mean survey duration was 5 minutes and 58 seconds; the median survey duration was 5 minutes.

**Dates of Data Collection:** Data were collected from December 22, 2023, to January 4, 2024.

**Sample Size:** We gathered survey responses from 1,250 respondents. After removing responses from a respondent who took the survey twice (i.e., we had 1,251 responses), we have a final sample of 1,249 respondents and responses.

**Whether and How the Data Were Weighted:** We do not use weights in our analyses.

**How the Data Were Processed and Procedures to Ensure Data Quality:** Our survey included an attention check. Per our pre-analysis plan, we do not exclude respondents who fail the attention check from our main analysis, but in a robustness check we do exclude such respondents and show that our results are robust to their exclusion. We do not exclude respondents based upon completion time. The CloudResearch Connect platform regularly engages in respondent quality checks, including conducting attention checks and ensuring that users are not participating from multiple accounts (Hartman et al. 2023). All survey responses record a participant’s unique CloudResearch ID; this allows us to ensure that users were not completing the survey more than once (and to remove one respondent who did; see the above Sample Size section). All responses were provided using the instrument outlined above. We do not employ manual or automated coding of responses beyond what is detailed in the replication code for this study (e.g., there were no open-ended questions).

**Screening Criteria and Process:** The survey was available to American adult participants on the CloudResearch Connect platform whose characteristics met our quota characteristics as described above in the Methods Used to Generate and Recruit the Sample section at the time they were on the platform.

**Study Stimuli:** There are no particular exhibits to report; we outline the full questionnaire and survey procedure in the Measurement Tools/Instruments section above.

**Dispositions or Response or Participation Rates:** This study utilized a non-probability sample recruited through CloudResearch. 2.95 percent of respondents who initially accepted the invitation to take our survey did not complete it, and 97.05 percent of respondents did complete it. However, because this is an online non-probability sample, the participation rate should not be interpreted as a response rate from a probability sample; this is a limitation of our sample.

**Sample Sizes:** As discussed above, our initial sample size included 1,250 respondents; after removing one respondent who took the survey twice, we have a final sample of 1,249 respondents. Each respondent evaluated two nominee profiles; this means that we have 2,498 evaluations in our full sample. In our main analyses (figure 1, figure 2a, and figure 2b), we employ our full sample. A small number of evaluations are dropped from these analyses if respondents did not answer the outcome question measuring support for the nominee (we have 2,494 evaluations where the respondent did answer this question). In various supplemental analyses and robustness checks, this full sample is occasionally subset: in Supplementary Material figure E.2, respondents who answered the middle “neither support nor oppose” option are dropped (resulting in 2,091 observations); in Supplementary Material figure E.4, one respondent (two observations) did not answer the ideology question and is dropped (resulting in 2,492 observations); in Supplementary Material figure E.5, we drop respondents who did not answer the respondent-level covariate questions (resulting in 2,476 observations); in Supplementary Material figure E.6, we subset to evaluations of the first nominee profile only (resulting in 1,247 observations); in Supplementary Material figure E.7, we exclude respondents who failed our attention check (resulting in 2,430 observations); in Supplementary Material figure E.8, one respondent (two observations) did not answer the identity questions and is dropped (resulting in 2,492 observations); in Supplementary Material figure E.10, we subset to nominee profiles that are cisgender and straight (resulting in 634 observations); in Supplementary Material figure E.11, the outcome variable is an additive legitimacy index and we only employ respondents who answered all five legitimacy outcome questions that we use to create this index (resulting in 2,480 observations).

**Measurement and Model Specification:** To replicate the variable creation and statistical modeling that generates the findings in the paper, please refer to the replication code for this study, available at https://doi.org/10.7910/DVN/CDLVDH. The code allows for full variable creation and the replication of all analyses that appear in the main text and the Supplementary Material.

**A General Statement Acknowledging Limitations of the Design and Data Collection:** We acknowledge limitations of our design and data collection. First, our respondents come from a non-probability online sample. Second, we ask respondents to consider hypothetical nominees under a particular presidency. Both limitations set possible scope conditions for the generalizability of our findings.
