# Supplementary material for: Ideological Cues, Partisanship, and Prejudice Against LGBTQ Judges
Source: Public Opin Q. 2026 Mar 3;90(1):218–37. doi: 10.1093/poq/nfaf064 (PMC13034043; doi:10.1093/poq/nfaf064)
Supplement: nfaf064_Supplementary_Data [file nfaf064_supplementary_data.pdf]

Supplementary Material

Ideological Cues, Partisanship, and Prejudice Against LGBTQ Judges

Andrew R. Stone and Tony Zirui Yang*

Contents

A AAPOR-Required Disclosure Elements **SM – 2**

B Survey Descriptive Statistics **SM – 9**

C Discussion of Pre-Analysis Plan **SM – 10**

D Full Empirical Results **SM – 11**

D.1 Support Full Results . . . . SM – 11
D.2 Double Penalty Full Results . . . . SM – 13
D.3 Penalty for Men and Women Nominees Full Results . . . . SM – 14

E Robustness Checks and Additional Analyses **SM – 16**

E.1 Support for Nominee, Five-Point Measure . . . . SM – 17
E.2 Support for Nominee, Dropping Middle Response Category . . . . SM – 18
E.3 Support for Nominee, Coding Leaners as Independents . . . . SM – 19
E.4 Support for Nominee, Results By Ideology . . . . SM – 20
E.5 Support for Nominee, Respondent-Level Controls . . . . SM – 21
E.6 Support for Nominee, First Profile Only . . . . SM – 22
E.7 Results Excluding Respondents Who Fail the Attention Check . . . . SM – 23
E.8 Results By Respondent LGBTQ Identity . . . . SM – 24
E.9 Results By Respondent Knowledge of Court . . . . SM – 25
E.10 Results for Just Cisgender, Straight Nominees . . . . SM – 26
E.11 Legitimacy Results . . . . SM – 27

F Additional Discussion of Argument and Findings **SM – 28**

F.1 Existing Scholarship on Prejudice . . . . SM – 28
F.2 Consideration of Generalizability of Findings . . . . SM – 28

*Andrew R. Stone (arstone@olemiss.edu) is an Assistant Professor, Department of Political Science, University of Mississippi. Tony Zirui Yang (zirui.yang@nuffield.ox.ac.uk) is an Assistant Professor of Political Science at Emory University and a Postdoctoral Prize Research Fellow in Politics at Nuffield College, University of Oxford.

A AAPOR-Required Disclosure Elements

Data Source and Data Collection Strategy

We employ survey data collected via a survey we administered on the CloudResearch Connect online platform.

Research Sponsor and Conductor

The research was sponsored by resources provided to one of the authors from their academic institution. The research was conducted by the authors.

Measurement Tools/Instruments

The survey began with an informed consent screen.
Then, respondents were asked four factual Supreme Court knowledge questions:

- Do you know if the U.S. Supreme Court can declare an act of Congress unconstitutional, or does it not have this power?
  - Can declare an act unconstitutional/Cannot declare an act unconstitutional/Don't know
- Some judges in the U.S. are elected; others are appointed. Do you happen to know if the Justices of the U.S. Supreme Court are elected or appointed?
  - Elected/Appointed/Some are elected and some are appointed/Don't know
- Some judges in the U.S. serve for a set number of years; others serve a life term. Do you happen to know whether the Justices of the U.S. Supreme Court serve for a set number of years or whether they serve a life term?
  - Set number of years/ Life term/ Some serve a set number of years and some serve a life term/ Don't know
- Which of the following people does not currently serve on the U.S. Supreme Court?
  - Clarence Thomas/Samuel Alito/Anthony Kennedy/Elena Kagan/Sonia Sotomayor/John Roberts

Then, respondents were presented with an attention check that read as follows:

- What news people watch affects their judgment on many issues. However, in this question, we only want to test whether you pay attention to the questions. Hence, regardless of what you are interested in, please choose Economic News and Sports News.

– Political News/Local News/International News/Economic News/News Interviews/ Investigative Journalism/Entertainment News/Technology News/Stock Market News/ Sports News/All of the above/None of the above

Then, respondents were presented with the first hypothetical nominee profile. They received the following introductory prompt and the profile, where each nominee had one of the randomly assigned characteristics displayed in Table A.1 below apart from the statement from Biden, which was randomly assigned to be shown to only half of the respondents:

- Suppose that next month, a vacancy arises on the U.S. Supreme Court and President Biden nominates the following individual to the Court:

TABLE A.1: Characteristics of Hypothetical Supreme Court Judges

**Attributes**	**Values**
Age	(a) 45; (b) 55; (c) 65
Race/Ethnicity	(a) Black; (b) Asian; (c) Hispanic
Law school	(a) Elite law school at an Ivy League university; (b) Well-regarded law school at a large public university; (c) Law school not ranked in the top 100 law schools
Current job	(a) U.S. district court judge; (b) Public defender; (c) Law professor at a top law school; (d) Corporate defense attorney
Political views	(a) Very liberal; (b) Liberal; (c) Somewhat liberal; (d) Moderate
Gender	(a) Man; (b) Transgender man; (c) Woman; (d) Transgender woman
Sexual orientation	(a) Straight; (b) Gay/lesbian
Statement from Biden (*Half of respondents*)	(a) "This nominee has an outstanding legal record and is well-qualified to serve on the federal judiciary."; (b) "This nominee will be a principled progressive voice on the federal judiciary."; (c) "This nominee will contribute to a diverse judiciary and make the federal judiciary look more like America."; (d) "This nominee will provide much-needed representation in the federal judiciary to a community historically underrepresented on the bench."

Note: One value from each attribute was randomly assigned to respondents for each hypothetical judge, apart from the Biden statement, which was randomly assigned to half of the respondents. If respondents were randomized into receiving a judge with gay or lesbian sexual orientation, the word displayed matched the judge's gender identity.

Then, respondents were asked to evaluate their support for the nominee:

- On a scale from strongly oppose to strongly support, where would you place your level of support for this nominee?
    - Strongly oppose/somewhat oppose/neither oppose nor support/somewhat support/strongly support

Then, respondents were asked to evaluate the legitimacy of the judiciary if the nominee were to be on the Court. They were first presented with the following prompt:

- Suppose this nominee was confirmed and began serving as a judge on the U.S. Supreme Court. Please indicate whether you would agree or disagree with the following, keeping in mind your evaluation of the nominee:

Then, respondents answered five legitimacy questions. All response options were a five-point measure of agreement (Strongly agree/somewhat agree/neither agree nor disagree/somewhat disagree/strongly disagree) The wording of the Supreme Court legitimacy questions are as follows:

- If the U.S. Supreme Court started making a lot of decisions that most people disagree with, it might be better to do away with the Supreme Court altogether.
- I would trust the U.S. Supreme Court to make decisions that are right for the country as a whole.
- I would support removing judges from their position on the U.S. Supreme Court if they consistently made decisions at odds with what a majority of the people want.
- The U.S. Supreme Court will have become too mixed up in politics.
- The U.S. Supreme Court will have become too independent and should be seriously reined in.

Then, respondents received a second nominee profile. They received the following prompt:

- Now, suppose that next month, a vacancy arises on the U.S. Supreme Court and President Biden nominates the following individual to the Court:

The survey then proceeded in the same manner as described above.

Finally, respondents were asked to answer questions measuring their traits and demographic information:

- How much school or college have you completed?
  - Some high school, or less/High school graduate or GED/Some college, no 4-year degree/College graduate/Post-graduate degree
- Generally speaking, do you think of yourself as a Republican, Democrat, Independent, or something else?
  - Republican/Democrat/Independent/Other
    - If Republican: Would you call yourself a strong Republican, or not a very strong Republican?
      - Strong Republican/Not very strong Republican
    - If Democrat: Would you call yourself a strong Democrat, or not a very strong Democrat?
      - Strong Democrat/Not very strong Democrat

        * If Independent or Other: Do you think of yourself as closer to the Republican or Democratic party?
            · Republican/Democratic/Neither

- How would you describe your political views?
    - Very liberal/Somewhat liberal/Moderate/Somewhat conservative/Very conservative
- Which best describes your total annual household income? If you're not sure, give an estimate.
    - Less than $25,000/$25,000 to $50,000/$50,000 to $75,000/$75,000 to $100,000/$100,000 to $200,000/$200,000 or more
- Which best describes your race? (Select all that apply)
    - White/Black or African American/Hispanic or Latino/Asian/American Indian or Alaska Native/Native Hawaiian or Pacific Islander/Other
- What sex were you assigned at birth, on your original birth certificate?
    - Male/Female
- What is your current gender identity?
    - Man/Woman/Transgender man/Transgender woman/ Do not identify as man, woman, or transgender
- Do you think of yourself as:
    - Straight/Gay or lesbian/Bisexual/ Do not identify as straight, gay or lesbian, or bisexual

Population Under Study

Our study population is American adults; we drew our study's participants from the pool of American adults active on the CloudResearch Connect platform at the time of our survey.

Methods Used to Generate and Recruit the Sample

The sample of 1,250 respondents comes from the pool of American adults active on the CloudResearch Connect platform at the time of our survey. This is a non-probability sample. The platform is an opt-in platform. We employed the CloudResearch built-in census match template to quota-target respondents to mirror the makeup of the U.S. population on age, race, ethnicity, and gender. These led us to quota target 625 men and 625 women; 275 people aged 18-29, 325 aged 30-44, 325 aged 45-59, and 325 aged 60-99; 200 individuals of Hispanic origin and 1050 individuals of non-Hispanic origin; and 975 White respondents, 175 Black respondents,

and 100 respondents of other races. We also quota sampled based upon respondent political party to secure 500 Democrats, 500 Republicans, and 250 other respondents. Respondents were paid $1.20 for completing the survey. Payment is provided through the CloudResearch platform; respondents can withdraw their earnings via PayPal, bank transfer, or Amazon gift cards.

Method(s) and Mode(s) of Data Collection

All survey responses were gathered on the web using Qualtrics. All surveys were conducted in English. The average survey duration was 5 minutes and 58 seconds; the median survey duration was 5 minutes.

Dates of Data Collection

Data were collected from December 22, 2023 to January 4, 2024.

Sample Size

We gathered survey responses from 1,250 respondents. After removing responses from a respondent who took the survey twice (i.e., we had 1,251 responses), we have a final sample of 1,249 respondents and responses.

Whether and How the Data Were Weighted

We do not use weights in our analyses.

How the Data Were Processed and Procedures to Ensure Data Quality

Our survey included an attention check. Per our pre-analysis plan, we do not exclude respondents who fail the attention check from our main analysis, but in a robustness check we do exclude such respondents and show that our results are robust to their exclusion. We do not exclude respondents based upon completion time. The CloudResearch Connect platform regularly engages in respondent quality checks including conducting attention checks and ensuring users are not participating from multiple accounts (Hartman et al. 2023). All survey responses record a participant's unique CloudResearch ID; this allows us to ensure that users were not completing the survey more than once (and to remove one respondent who did; see the above Sample Size section). All responses were provided using the instrument outlined above. We do not employ manual or automated coding of responses beyond what is detailed in the replication code for this study (e.g., there were no open-ended questions).

Screening Criteria and Process

The survey was available to American adult participants on the CloudResearch Connect platform whose characteristics met our quota characteristics as described above in the Methods Used to Generate and Recruit the Sample section at the time they were on the platform.

Study Stimuli

There are no particular exhibits to report; we outline the full questionnaire and survey procedure in the Measurement Tools/Instruments section above.

Dispositions or Response or Participation Rates

This study utilized a non-probability sample recruited through CloudResearch. 2.95 percent of respondents who initially accepted the invitation to take our survey did not complete it and 97.05 percent of respondents did complete it. However, because this is an online non-probability sample, the participation rate should not be interpreted as a response rate from a probability sample; this is a limitation of our sample.

Sample Sizes

As discussed above, our initial sample size included 1,250 respondents; after removing one respondent who took the survey twice, we have a final sample of 1,249 respondents. Each respondent evaluated two nominee profiles; this means that we have 2,498 evaluations in our full sample. In our main analyses (Figure 1, Figure 2a, and Figure 2b), we employ our full sample. A small number of evaluations are dropped from these analyses if respondents did not answer the outcome question measuring support for the nominee (we have 2,494 evaluations where the respondent did answer this question). In various supplemental analyses and robustness checks, this full sample is occasionally subset: in Figure E.2 respondents who answered the middle "neither support nor oppose" option are dropped (resulting in 2,091 observations); in Figure E.4, one respondent (two observations) did not answer the ideology question and is dropped (resulting in 2,492 observations); in Figure E.5 we drop respondents who did not answer the respondent-level covariate questions (resulting in 2,476 observations); in Figure E.6 we subset to evaluations of the first nominee profile only (resulting in 1,247 observations); in Figure E.7 we exclude respondents who failed our attention check (resulting in 2,430 observations); in Figure E.8 one respondent (two observations) did not answer the identity questions and is dropped (resulting in 2,492 observations); in Figure E.10 we subset to nominee profiles that are cisgender and straight (resulting in 634 observations); in Figure E.11 the outcome variable is an additive legitimacy index and we only employ respondents who answered all five legitimacy outcome questions that we use to create this index (resulting in 2,480 observations).

Measurement and Model Specification

To replicate the variable creation and statistical modeling that generates the findings in the paper, please refer to the replication code for this study. The code allows for full variable creation and the replication of all analyses that appear in the main text and Supplementary Material.

A General Statement Acknowledging Limitations of the Design and Data Collection

We acknowledge limitations of our design and data collection. First, our respondents come from a non-probability online sample. Second, we ask respondents to consider hypothetical

nominees under a particular presidency. Both limitations set possible scope conditions for the generalizability of our findings.

B Survey Descriptive Statistics

TABLE B.1: Descriptive Statistics: CloudResearch Survey

Category	Proportion	Category	Proportion
**Gender**		**Education**	
Man	.497	Some high school, or less	.006
Woman	.496	High school graduate or GED	.116
Transgender	.005	Some college, no 4-year degree	.300
Other	.002	College graduate	.422
		Post-graduate degree	.156
**Race**		**Income**	
White	.655	Less than $25,000	.134
Black	.127	$25,000 to $50,000	.245
Hispanic or Latina/o	.127	$50,000 to $75,000	.230
Asian/Native Hawaiian/Pacific Islander	.046	$75,000 to $100,000	.171
Native American/Alaskan	.002	$100,000 to $200,000	.184
Multiple racial groups	.034	$200,000 or more	.036
Other racial group	.003		
**Partisanship**		**Ideology**	
Democrat	.487	Liberal	.417
Republican	.422	Moderate	.204
Independent	.091	Conservative	.379
**Sexuality**			
Straight	.892		
Gay, lesbian, or bisexual	.099		
Other	.010		

Note: Cell entries indicate unweighted sample proportions for each demographic and political category. Proportions may not add to 1 due to rounding or non-response. Leaners are coded as partisans. N = 1,249.

C Discussion of Pre-Analysis Plan

Our pre-analysis plan is available at https://aspredicted.org/blind.php?x=ZPZ_K41. We pre-registered five specific hypotheses: three we find clear evidence for and two we do not find clear evidence for. In the discussion of Figure 1, we show that individuals react less favorably to LGBTQ judges than non-LGBTQ judges (in line with our H1) and less favorably to transgender than gay/lesbian judges (our H2). In the discussion of Figure 2a, we show evidence of a double penalty for transgender and gay/lesbian judges (our H3). However, in Figure 1 we do not find stronger effects for Republicans than other respondents (our H4). In Figure D.2, we show that messaging from partisan elites (Biden) has the directional effects we hypothesized (positive for copartisans and negative for outpartisans), but these effects are not statistically distinguishable from zero (our H5).

We conducted our survey as specified in the plan. We measure our variables as specified in the plan. We present the analyses discussed in the plan either in the main text or the Supplementary Material (see Supplementary Material Sections D and E) using the cjoint package in R (Hainmueller, Hopkins, and Yamamoto 2014). Additional analyses that do not appear in the pre-analysis plan were added based upon helpful feedback provided when circulating the paper.

D Full Empirical Results

D.1 Support Full Results

FIGURE D.1: Nominee Traits and Support for Supreme Court Nominees (Aggregate Results)

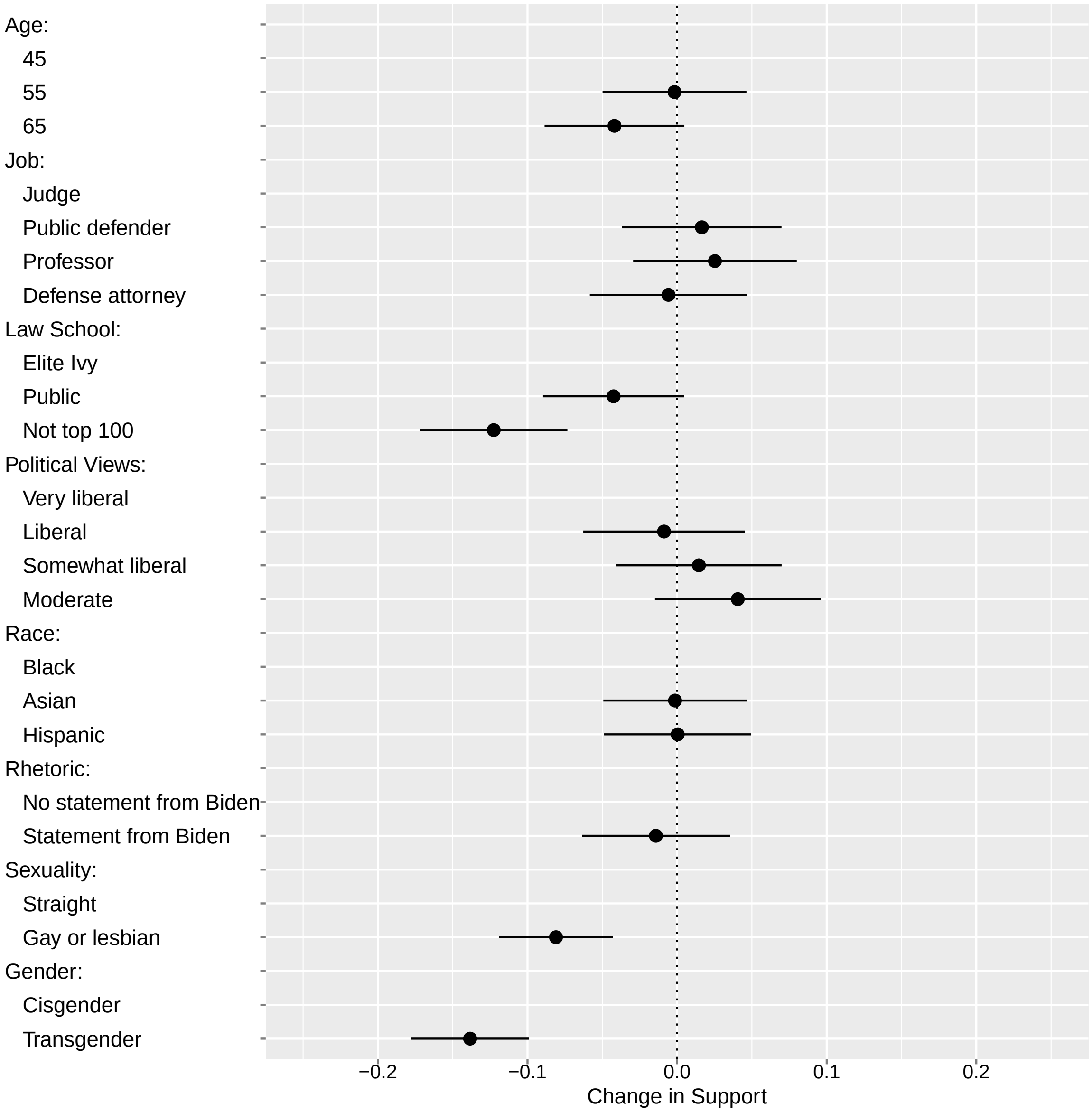

Note: Outcome is a binary measure of support for Supreme Court nominees. Bars represent the Average Marginal Component Effect with 95 percent confidence intervals for each of the varying attributes in our conjoint analysis; standard errors clustered by respondent.

FIGURE D.2: Nominee Traits and Support for Supreme Court Nominees (Results By Party)

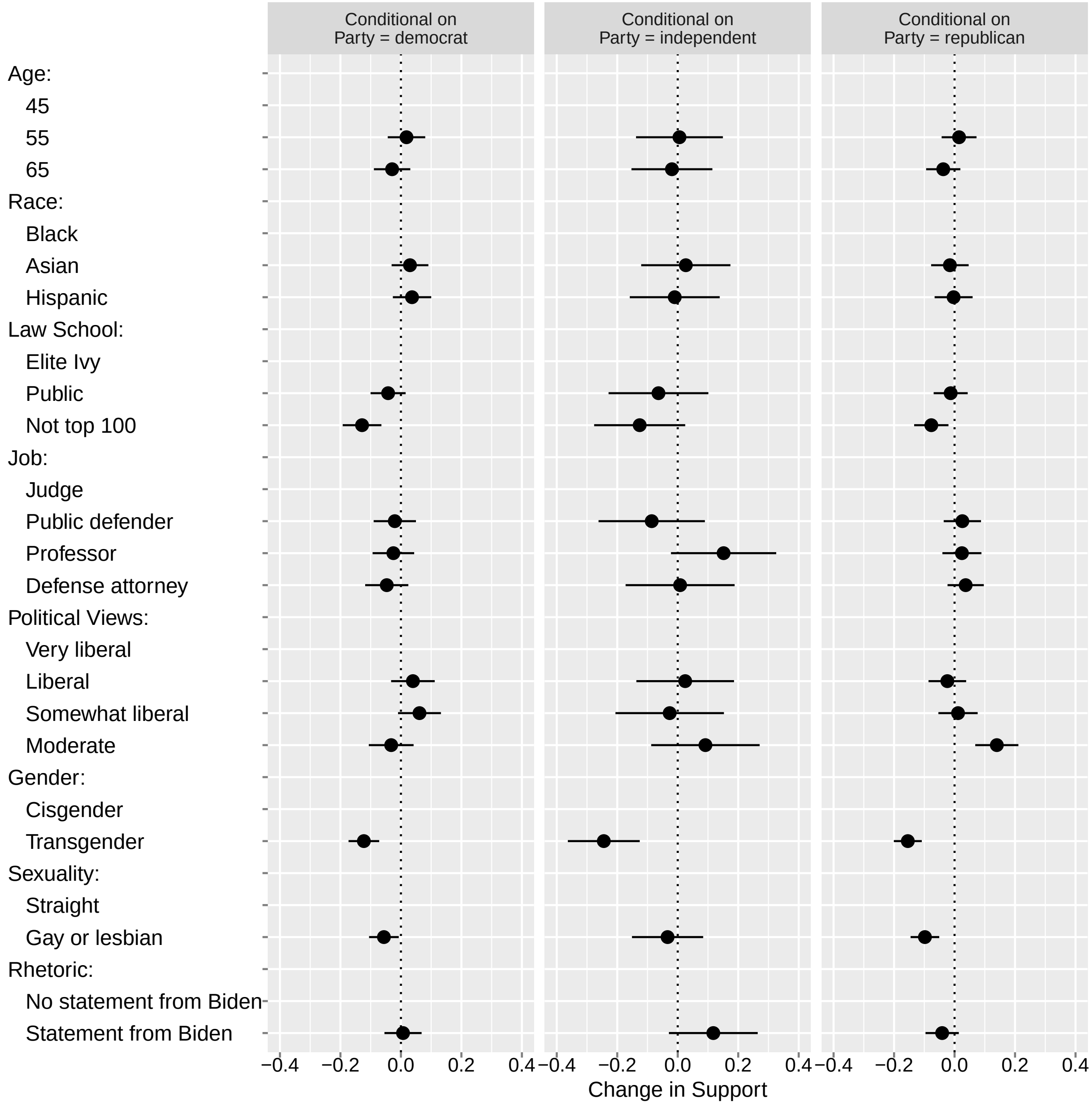

Note: Outcome is a binary measure of support for Supreme Court nominees. Bars represent the Average Marginal Component Effect with 95 percent confidence intervals for each of the varying attributes in our conjoint analysis; standard errors clustered by respondent.

D.2 Double Penalty Full Results

FIGURE D.3: Double Penalty of Transgender and Gay Nominees

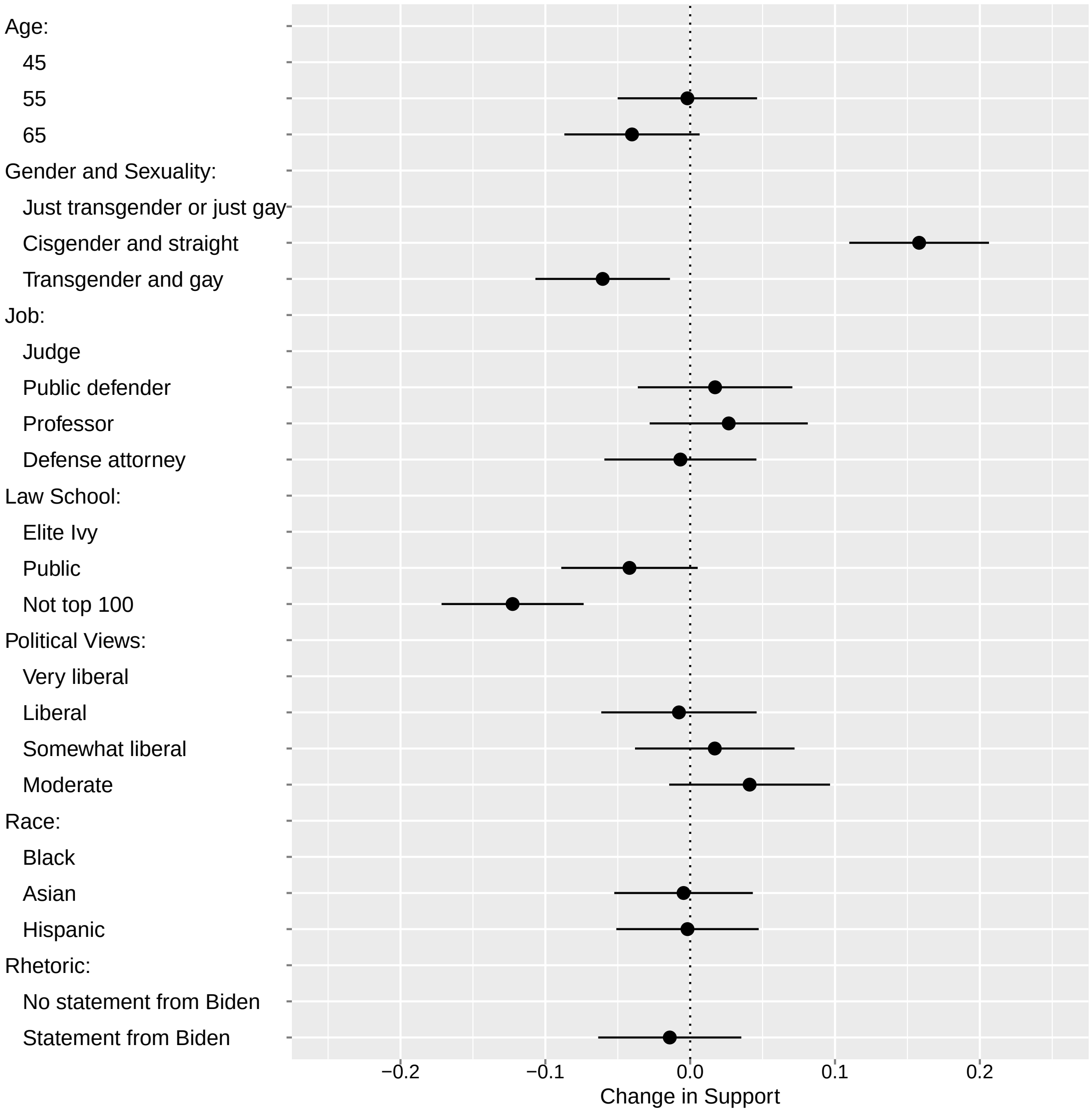

Note: Outcome is a binary measure of support for Supreme Court nominees. Bars represent the Average Marginal Component Effect with 95 percent confidence intervals for each of the varying attributes in our conjoint analysis; standard errors clustered by respondent. Treatment effects plotted for judges who are transgender and gay/lesbian or judges who are cisgender and straight as compared to judges who are transgender or gay/lesbian (baseline).

D.3 Penalty for Men and Women Nominees Full Results

FIGURE D.4: Nominee Traits and Support (Men Nominees Only)

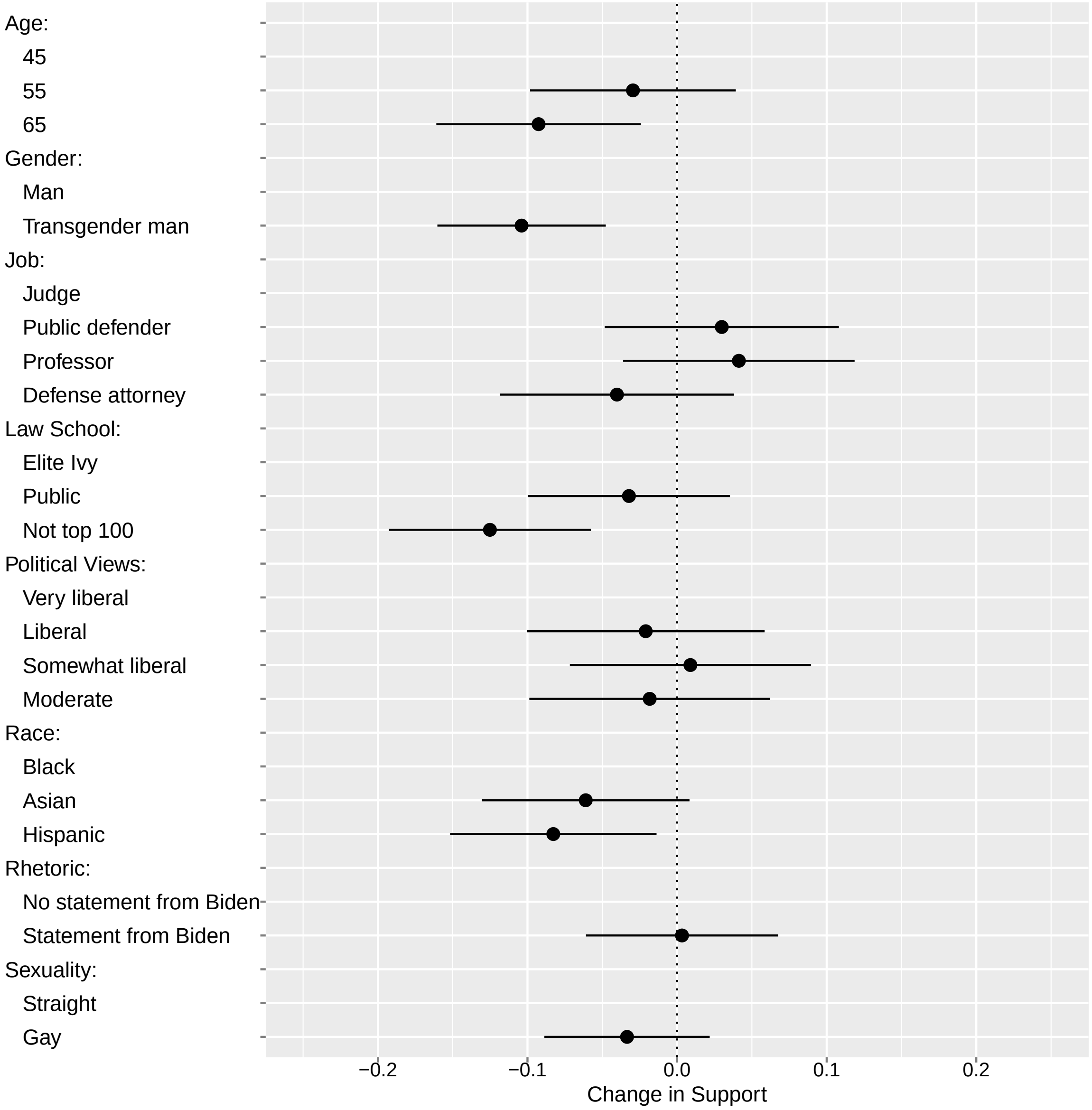

Note: Outcome is a binary measure of support for Supreme Court nominees. Bars represent the Average Marginal Component Effect with 95 percent confidence intervals for each of the varying attributes in our conjoint analysis; standard errors clustered by respondent. Data are subset to nominee profiles who are men.

FIGURE D.5: Nominee Traits and Support (Women Nominees Only)

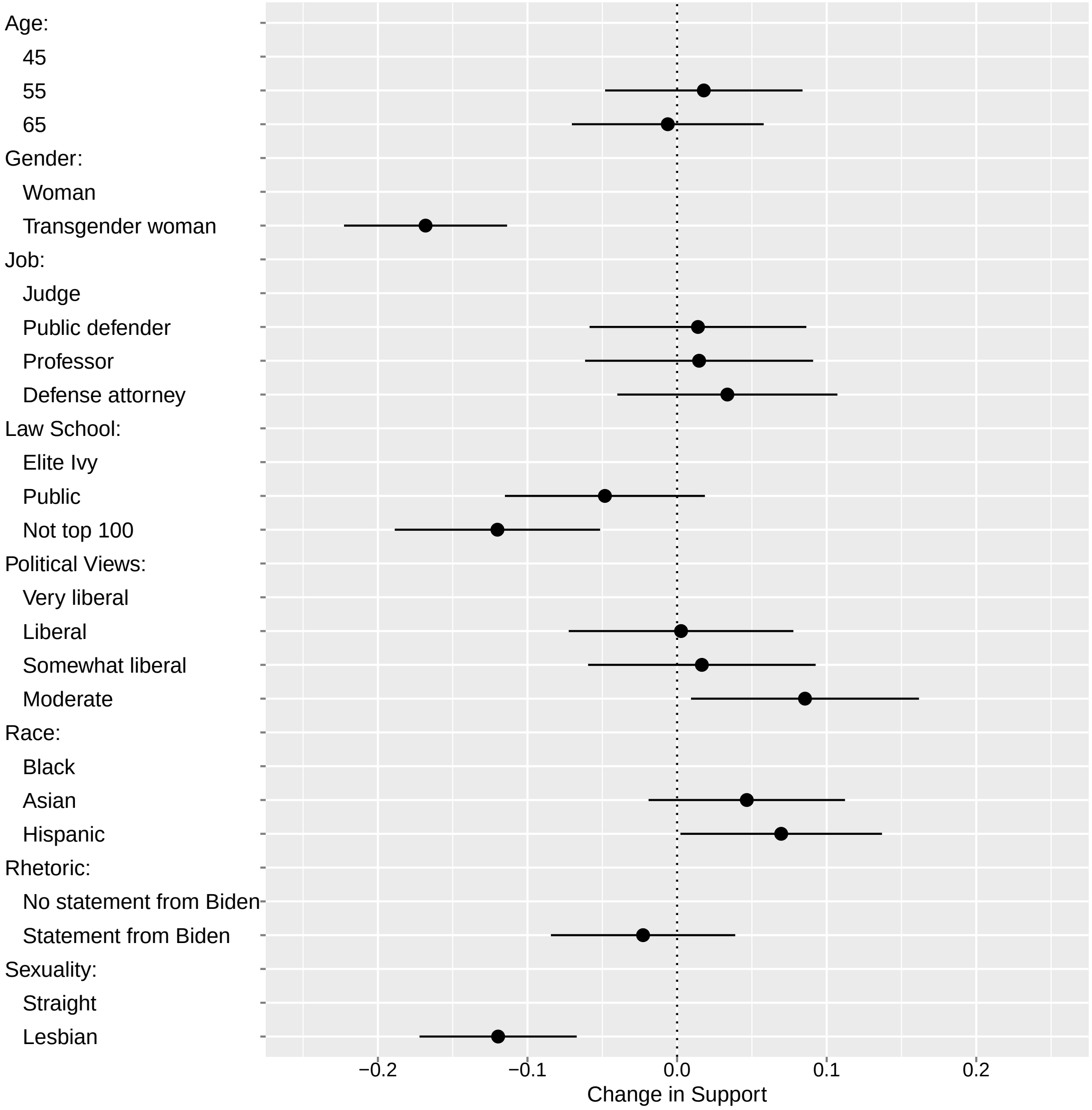

Note: Outcome is a binary measure of support for Supreme Court nominees. Bars represent the Average Marginal Component Effect with 95 percent confidence intervals for each of the varying attributes in our conjoint analysis; standard errors clustered by respondent. Data are subset to nominee profiles who are women.

E Robustness Checks and Additional Analyses

In this section, we report the results from a variety of robustness checks and additional analyses. In sum, our robustness checks (Figures E.1 to E.7) are consistent with the conclusions we draw in our main analyses. Our results are consistent when using a five-point support variable (Figure E.1), dropping "neither oppose nor support" respondents (Figure E.2), coding leaners as independents (Figure E.3), using respondent ideology (Figure E.4), controlling for respondent characteristics (Figure E.5), and limiting to first profile evaluations (Figure E.6) and to respondents who passed our attention check (Figure E.7).

Our additional analyses build upon our main text analyses. Figure E.8 shows that our results are driven by non-LGBTQ respondents; this aligns with research that shows Americans value judges who share their descriptive traits. Figure E.9 shows that our results are generally similar across levels of respondent Court knowledge; this speaks to scholarship on the role Court knowledge plays in shaping attitudes toward the judiciary (Gibson and Caldeira 2009). Figure E.10 reports the results from our analysis of just cisgender, straight nominee profiles that we discuss in the main text footnote 6. Figure E.11 shows that the effects of LGBTQ traits on respondent evaluations of Court legitimacy are of smaller magnitude and more limited statistical significance compared with support for individual nominees.

E.1 Support for Nominee, Five-Point Measure

FIGURE E.1: Effect of Transgender and Gay Nominees on Support (Five-Point Measure)

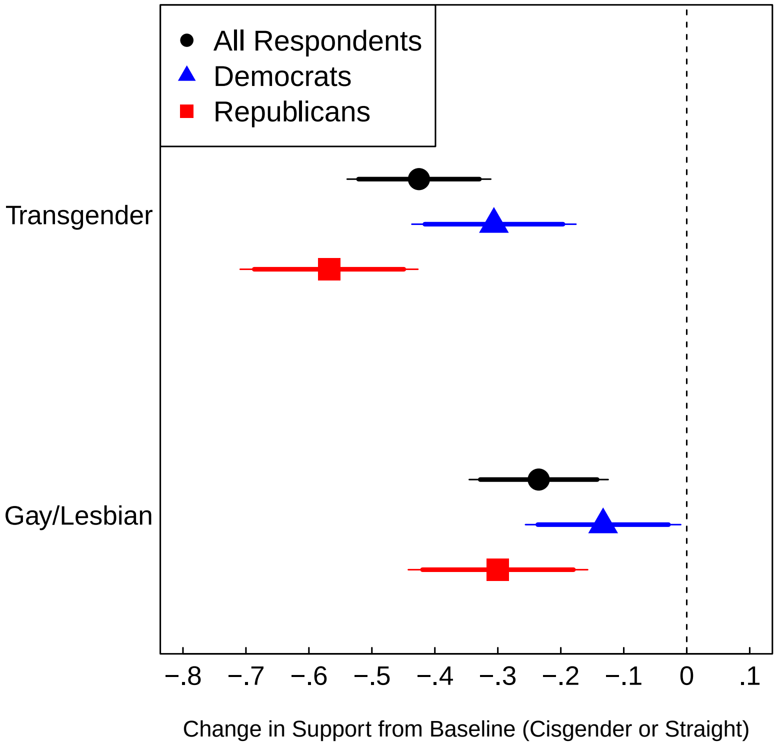

Note: Figure shows the estimated treatment effect (AMCE) on judge support (measured on a five-point scale) of a nominee's transgender or gay/lesbian identity (as compared to the baseline of cisgender or straight). Separate results are presented for all respondents, Democrats, and Republicans. 90 and 95 percent confidence intervals plotted for each estimate; standard errors clustered by respondent.

E.2 Support for Nominee, Dropping Middle Response Category

FIGURE E.2: Effect of Transgender and Gay Nominees on Support (Dropping Middle Response Category)

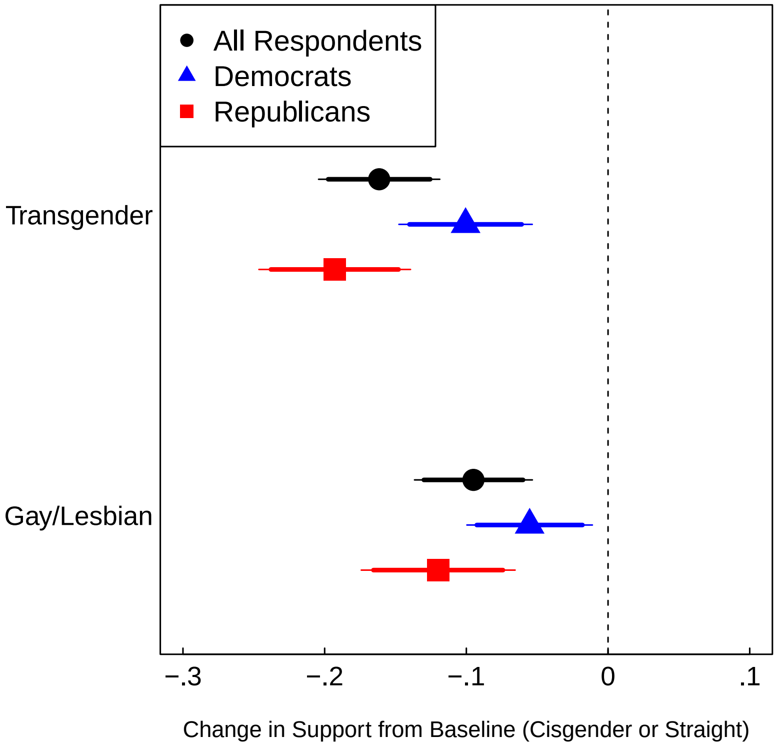

Note: Figure shows the estimated treatment effect (AMCE) on judge support of a nominee's transgender or gay/lesbian identity (as compared to the baseline of cisgender or straight). Respondents who answered "neither oppose nor support" to the support outcome question are dropped from the analysis. Separate results are presented for all respondents, Democrats, and Republicans. 90 and 95 percent confidence intervals plotted for each estimate; standard errors clustered by respondent.

E.3 Support for Nominee, Coding Leaners as Independents

FIGURE E.3: Effect of Transgender and Gay Nominees on Support (Leaners as Independents)

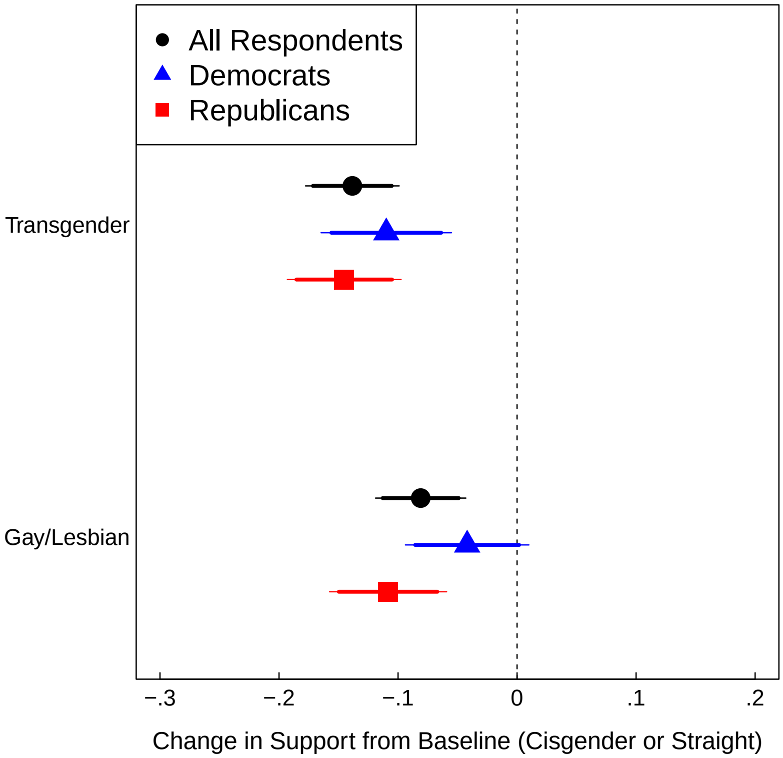

Note: Figure shows the estimated treatment effect (AMCE) on judge support of a nominee's transgender or gay/lesbian identity (as compared to the baseline of cisgender or straight). Leaners are coded as independents. Separate results are presented for all respondents, Democrats, and Republicans. 90 and 95 percent confidence intervals plotted for each estimate; standard errors clustered by respondent.

E.4 Support for Nominee, Results By Ideology

FIGURE E.4: Effect of Transgender and Gay Nominees on Support (Results by Ideology)

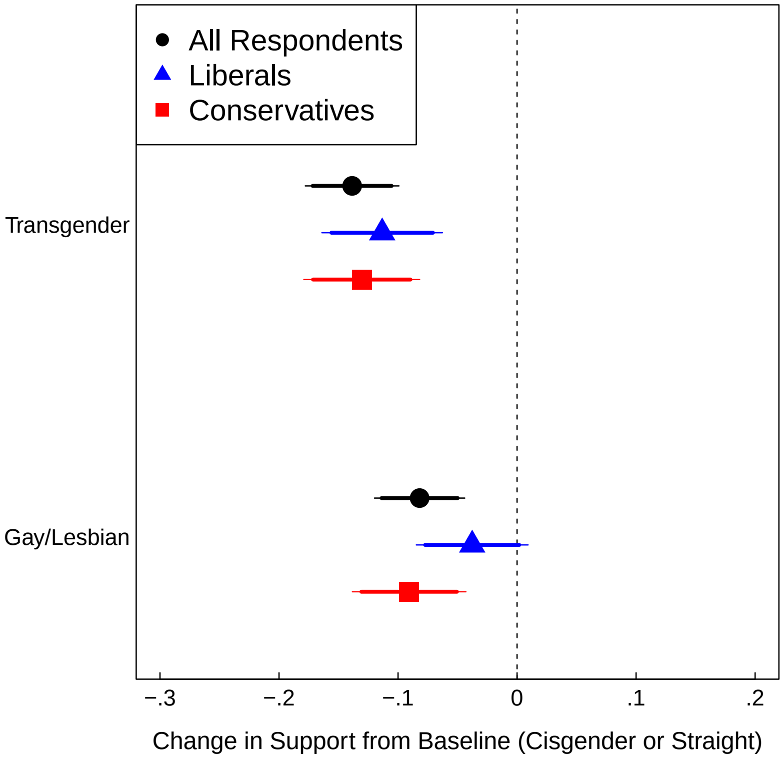

Note: Figure shows the estimated treatment effect (AMCE) on judge support of a nominee's transgender or gay/lesbian identity (as compared to the baseline of cisgender or straight). Separate results are presented for all respondents, liberals, and conservatives. 90 and 95 percent confidence intervals plotted for each estimate; standard errors clustered by respondent.

E.5 Support for Nominee, Respondent-Level Controls

FIGURE E.5: Effect of Transgender and Gay Nominees on Support (Respondent-Level Controls)

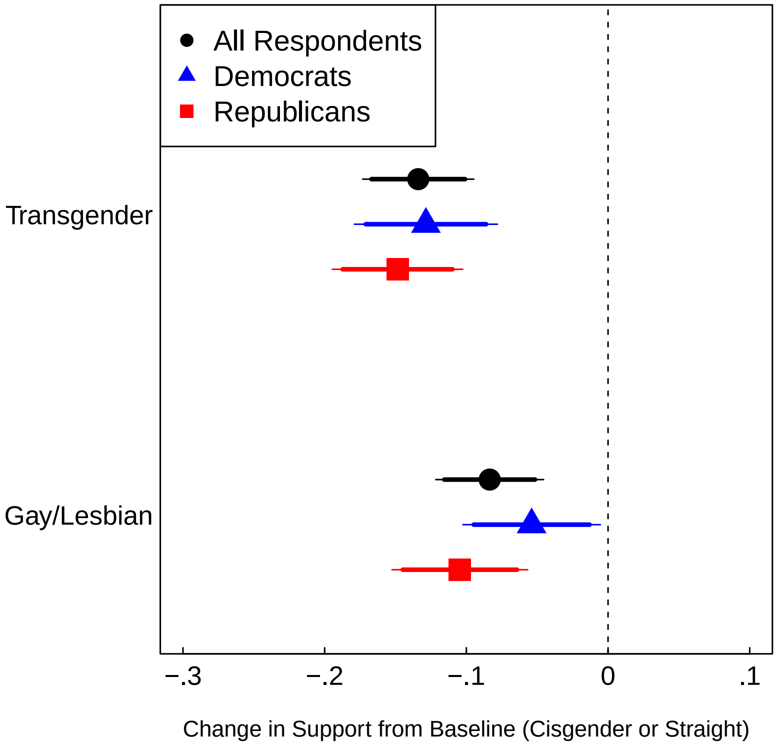

Note: Figure shows the estimated treatment effect (AMCE) on judge support of a nominee's transgender or gay/lesbian identity (as compared to the baseline of cisgender or straight). Analysis controls for respondent race (White/non-White), income (over $75,000/not), college degree, and gender (man/not). Separate results are presented for all respondents, Democrats, and Republicans. 90 and 95 percent confidence intervals plotted for each estimate; standard errors clustered by respondent.

E.6 Support for Nominee, First Profile Only

FIGURE E.6: Effect of Transgender and Gay Nominees on Support (First Profile Only)

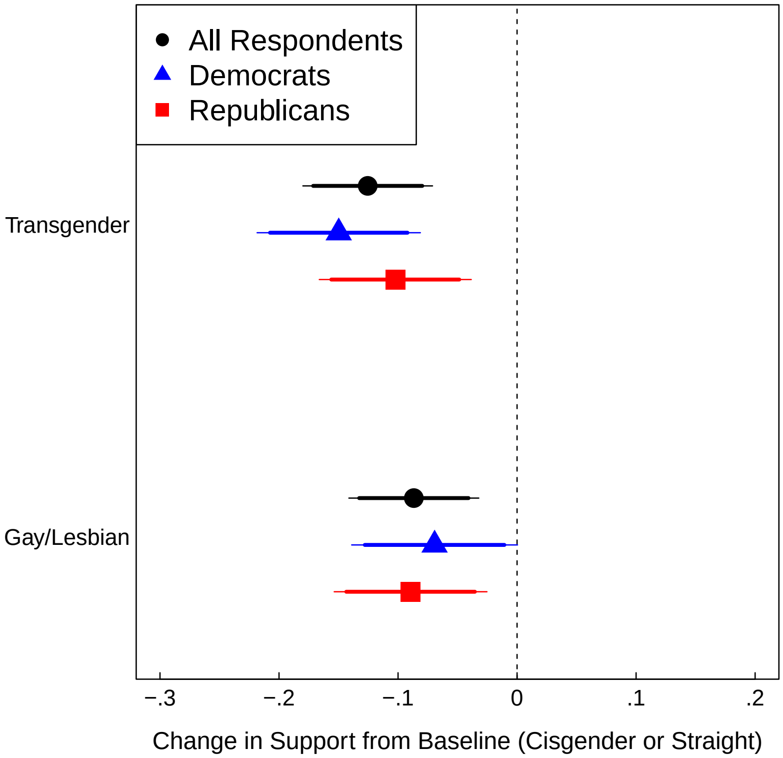

Note: Figure shows the estimated treatment effect (AMCE) on judge support of a nominee's transgender or gay/lesbian identity (as compared to the baseline of cisgender or straight). Results are subset to the first profile a respondent evaluated. Separate results are presented for all respondents, Democrats, and Republicans. 90 and 95 percent confidence intervals plotted for each estimate.

E.7 Results Excluding Respondents Who Fail the Attention Check

FIGURE E.7: Effect of Transgender and Gay Nominees on Support (Dropping Inattentive Respondents)

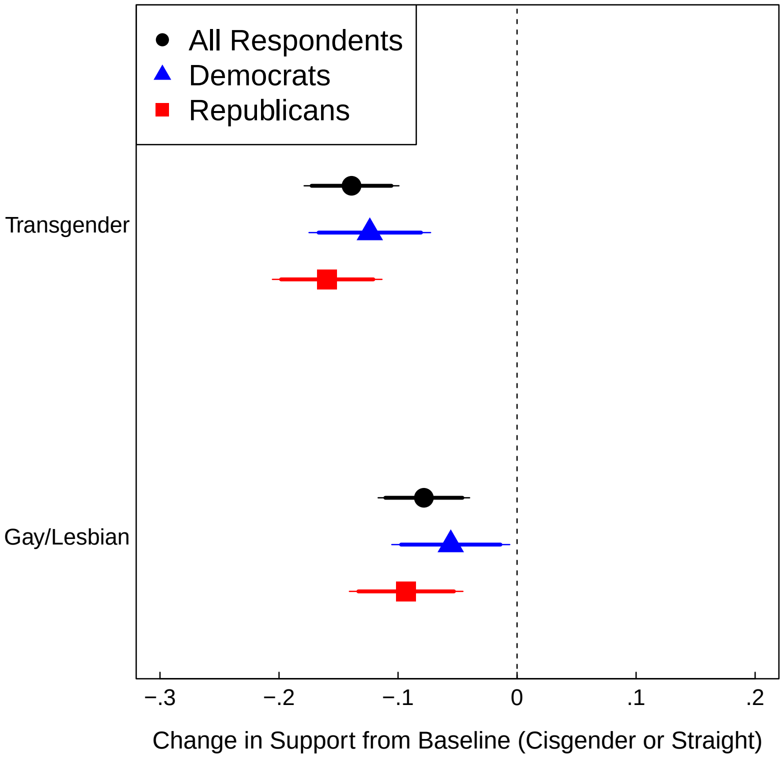

Note: Figure shows the estimated treatment effect (AMCE) on judge support of a nominee's transgender or gay/lesbian identity (as compared to the baseline of cisgender or straight). The 2.6 percent of respondents who fail the attention check are dropped from the analysis. Separate results are presented for all respondents, Democrats, and Republicans. 90 and 95 percent confidence intervals plotted for each estimate; standard errors clustered by respondent.

E.8 Results By Respondent LGBTQ Identity

FIGURE E.8: Effect of Transgender and Gay Nominees on Support (Results by Respondent LGBTQ Traits)

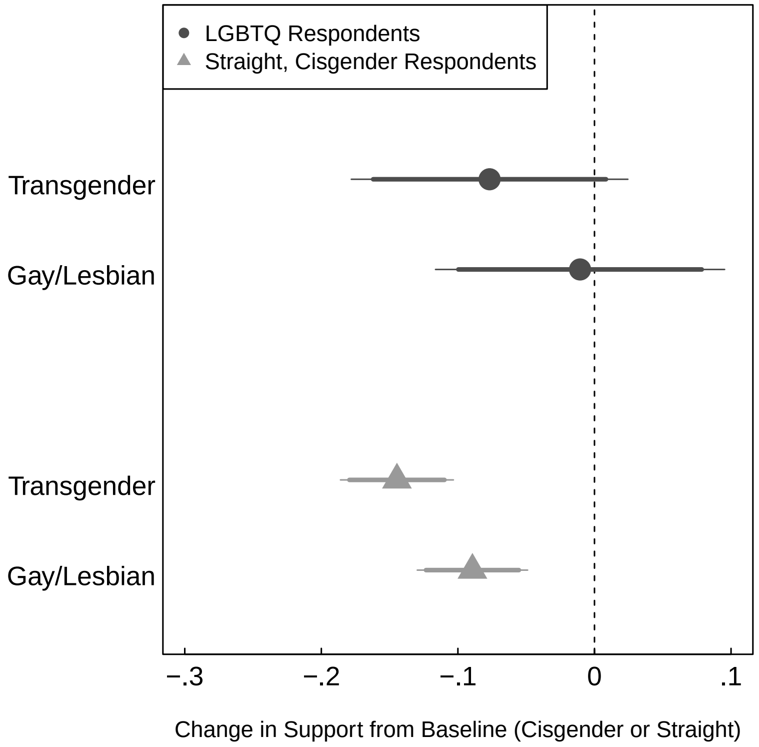

Note: Figure shows the estimated treatment effect (AMCE) on judge support of a nominee's transgender or gay/lesbian identity (as compared to the baseline of cisgender or straight). Separate results are presented for respondents who have LGBTQ traits (top points) or respondents who are straight and cisgender (bottom points). 90 and 95 percent confidence intervals plotted for each estimate; standard errors clustered by respondent.

E.9 Results By Respondent Knowledge of Court

FIGURE E.9: Effect of Transgender and Gay Nominees on Support (Results by Respondent Knowledge of Court)

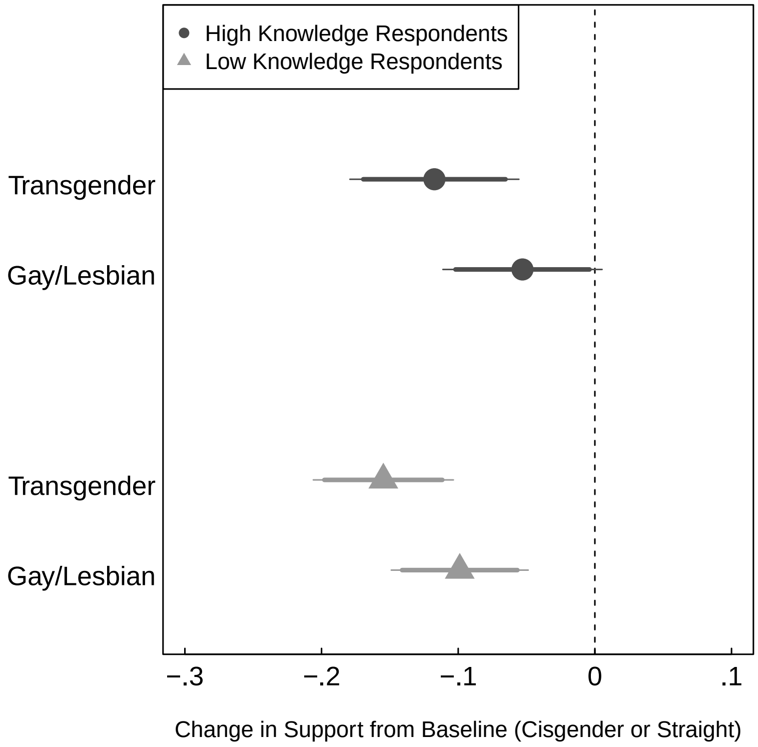

Note: Figure shows the estimated treatment effect (AMCE) on judge support of a nominee's transgender or gay/lesbian identity (as compared to the baseline of cisgender or straight). Separate results are presented for respondents who have high Court knowledge (4 factual questions correct, 44 percent of respondents; top points) or respondents who are low knowledge (0-3 factual questions correct, 56 percent of respondents; bottom points). Knowledge questions are presented in Supplementary Material Section A. Respondents who answered "don't know" or did not answer a knowledge question were coded as a 0 on knowledge for that question. 90 and 95 percent confidence intervals plotted for each estimate; standard errors clustered by respondent.

E.10 Results for Just Cisgender, Straight Nominees

FIGURE E.10: Effect of Gender on Support (Just Cisgender, Straight Nominees)

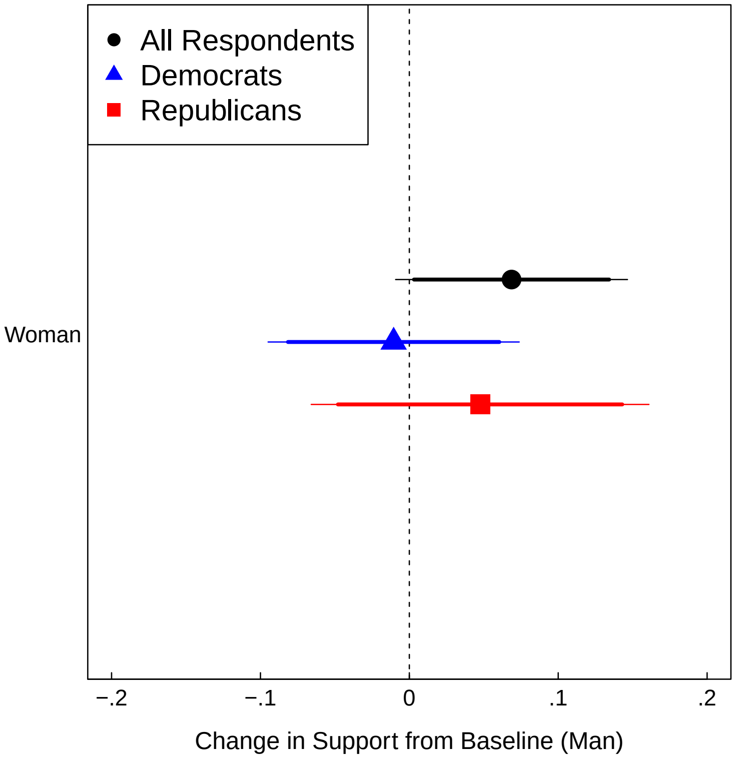

Note: Figure shows the estimated treatment effect (AMCE) on judge support of a nominee being a woman (as compared to the baseline of man). Data are subset to include only nominee profiles where the nominee is straight and cisgender. 90 and 95 percent confidence intervals plotted for each estimate; standard errors clustered by respondent.

E.11 Legitimacy Results

Figure E.11 illustrates that the effects of LGBTQ nominees on Court legitimacy (as measured via a five-question additive battery rescaled to range from 0-1 using the questions presented in Supplementary Material Section A) are of smaller magnitude and more limited statistical significance compared with support for individual nominees. Nevertheless, Republicans lower their evaluations of Court legitimacy by 8.8 percentage points (p<0.001) in response to evaluating a transgender judge and by 3.2 percentage points (p=0.079) for a gay or lesbian judge; we find no distinguishable effects for Democrats. These results suggest that, in addition to the negative impact on the support of individual nominees, diversifying the Court may provoke backlash against the institution as a whole.

FIGURE E.11: Effect of Transgender and Gay Nominees on Court Legitimacy

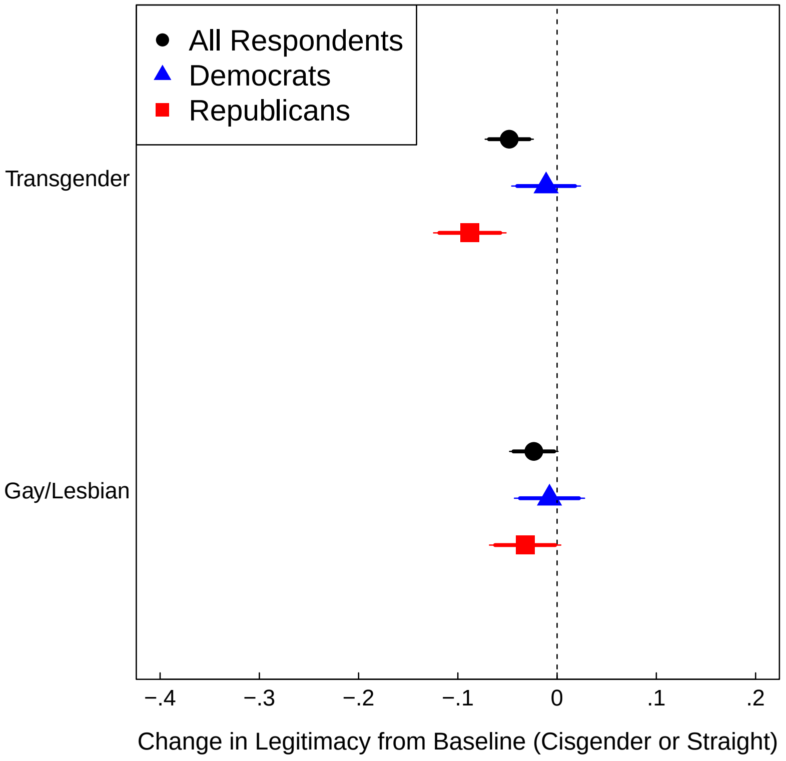

Note: Figure shows the estimated treatment effect (AMCE) of a nominee's transgender or gay/lesbian identity (as compared to the baseline of cisgender or straight) on evaluations of Court legitimacy (a five-question battery rescaled to range from 0-1) for all respondents, Democrats, and Republicans. 90 and 95 percent confidence intervals plotted for each estimate; standard errors clustered by respondent.

F Additional Discussion of Argument and Findings

F.1 Existing Scholarship on Prejudice

Existing scholarship is suggestive of the possibility that individuals across partisan identities may exhibit bias against LGBTQ individuals after accounting for the ideological cues LGBTQ identity can send. Consequently, when ideological instrumental incentives are accounted for, liberals may not be as supportive of LGBTQ politicians and judges as existing studies suggest. Research illustrates that racial prejudice appears across the political spectrum and plays a role in shaping political attitudes. For example, Hooghe and Dassonneville (2018) shows that racial resentment explains a voter's likelihood of voting for Trump, even after accounting for partisanship and ideology; these results emerge among both Republicans and Democrats. Further, Krupnikov and Piston (2015) show that racial resentment is associated with decreased turnout in the 2008 presidential election among strong Democrats. As the ideological views of presidential candidates are clear to voters, this suggests that after accounting for ideology, prejudice can emerge among voters that may be expected to support minority candidates on ideological grounds.

F.2 Consideration of Generalizability of Findings

We focus on public attitudes toward LGBTQ judicial nominees in this paper. Future studies should investigate how our findings for judges compare to other politicians such as legislators, executives, or bureaucrats. Such a study would speak to how our theoretical argument about shared ideology as an instrumental reason why individuals may support or oppose a LGBTQ politician generalize to other political contexts.

As Americans demand policy representation from their judges (Bartels and Johnston 2012) and contemporary American Supreme Court nominations and attitudes toward the Court are relatively polarized (Cameron and Kastellec 2023; Levendusky et al. 2024), we may expect muted

differences across contexts. Alternatively, Americans hold legalistic expectations of judge behavior that render the judiciary distinct from the other branches (Rivero and Stone 2025), this may generate differential effects across institutions.

References

Bartels, Brandon L, and Christopher D Johnston. 2012. "Political Justice? Perceptions of Politicization and Public Preferences Toward the Supreme Court Appointment Process." *Public Opinion Quarterly* 76(1): 105–116.

Cameron, Charles M, and Jonathan P Kastellec. 2023. *Making the Supreme Court: The Politics of Appointments, 1930-2020*. Oxford University Press.

Gibson, James L, and Gregory A Caldeira. 2009. "Knowing the Supreme Court? A reconsideration of public ignorance of the high court." *The Journal of Politics* 71(2): 429–441.

Hainmueller, Jens, Dan Hopkins, and Teppei Yamamoto. 2014. *cjoint: Causal Inference in Conjoint Analysis: Understanding Multi-Dimensional Choices via Stated Preference Experiments*.

Hartman, Rachel, Aaron J. Moss, Shalom N. Jaffe, Cheskie Rosenzweig, Leib Litman, and Jonathan Robinson. 2023. "Introducing Connect by CloudResearch: Advancing Online Participant Recruitment in the Digital Age." *PsyArXiv* https://doi.org/10.31234/osf.io/ksgyr.

Hooghe, Marc, and Ruth Dassonneville. 2018. "Explaining the Trump vote: The effect of racist resentment and anti-immigrant sentiments." *PS: Political Science & Politics* 51(3): 528–534.

Krupnikov, Yanna, and Spencer Piston. 2015. "Racial prejudice, partisanship, and White turnout in elections with Black candidates." *Political Behavior* 37: 397–418.

Levendusky, Matthew, Shawn Patterson Jr, Michele Margolis, Josh Pasek, Kenneth Winneg, and Kathleen H Jamieson. 2024. "Has the Supreme Court become just another political branch? Public perceptions of court approval and legitimacy in a post-Dobbs world." *Science Advances* 10(10): eadk9590.

Rivero, Albert H, and Andrew R Stone. 2025. "The American public's attitudes over how judges use legal principles to make decisions." *Political Science Research and Methods* 1: 167–182.
